# Representational similarity analysis reveals cue-independent spatial representations for landmarks and self-motion cues in human retrosplenial cortex

**DOI:** 10.1162/imag_a_00516

**Published:** 2025-03-24

**Authors:** Xiaoli Chen, Ziwei Wei, Thomas Wolbers

**Affiliations:** Department of Psychology and Behavioral Sciences, Zhejiang University, Hangzhou, China; German Center for Neurodegenerative Diseases (DZNE), Magdeburg, Germany; Department of Neurology, Otto-von-Guericke University, Magdeburg, Germany; Center for Behavioral Brain Sciences (CBBS), Otto-von-Guericke University, Magdeburg, Germany

**Keywords:** spatial navigation, fMRI, retrosplenial cortex, representational similarity analysis, path integration, landmark

## Abstract

It is a fundamental question in the spatial navigation literature how different spatial cues are unified to form a coherent spatial map of the space. Landmarks and self-motion cues are two major spatial cue types, which recruit relatively independent cognitive processes that dynamically interact with each other during navigation. In our previous studies, we developed two novel memory-dependent paradigms to contrast visual landmarks and visual self-motion cues in the desktop virtual reality environment. Participants visited the four test locations arranged evenly along a linear track in predetermined sequences. While at each test location, they performed a spatial judgment relying on memory. Using ultra-high field fMRI at 7 Tesla, we found that the human entorhinal cortex (EC) and retrosplenial cortex (RSC) exhibited cue-specific location-based spatial representations in the form of fMRI adaptation (fMRIa), meaning that the closer the two successively visited locations were to each other, the greater the suppression in the brain activation. In the current study, we re-analyzed the same fMRI datasets from our previous studies by performing the representational similarity analysis (RSA), an approach complementary to the fMRIa analysis in assessing neural representations. RSA’s rationale is that the closer two locations are to each other in the space, the more similar multi-voxel patterns of brain activation they should elicit. The results showed that RSC contained RSA-based neural representations of spatial locations for both landmarks and self-motion cues, which were overall driven by subjective response (participant’s self-reported location) instead of objective location (participant’s actual location). These representations were generalizable between the two cue types in terms of response, indicating cue-independent spatial representations. Combined with our previous finding of cue-specific fMRIa-based spatial representations in RSC, our study demonstrates the coexistence of cue-specific and cue-independent spatial representations in RSC. Our findings suggest that RSC plays a crucial role in unifying various spatial sensory inputs into coherent spatial representations, supporting memory-oriented navigation behavior.

## 1 Introduction

Spatial navigation is a fundamental ability for any mobile organism. A prominent feature of spatial navigation is that information about one’s position and orientation can typically be derived from a multitude of spatial cues, including static cues provided by the environment and dynamic self-motion cues. Despite the diversity of spatial information utilized, we usually perceive a unified spatial space during navigation. Therefore, a critical question is how a navigator synthesizes the array of spatial cues to construct a coherent spatial representation of the space. Understanding this cue unification process is paramount for unraveling the mechanisms governing the formation of cognitive maps, mental representations of space that enable flexible navigation behavior.

A major classification is made between landmarks and self-motion cues, two fundamental types of spatial information for navigation (Etienne et al., 1996). Navigation with self-motion cues—such as proprioceptive inputs, vestibular signals, and optic flow—requires continuous integration of self-movement to determine one’s location and direction, a process known as path integration (Etienne & Jeffery, 2004;Mittelstaedt & Mittelstaedt, 1980). In contrast, landmarks are prominent environmental features that provide direct spatial information. It is frequently observed that navigation relying on self-motion cues and landmarks involves distinct cognitive processes. For example, past studies have shown that varying the quality of landmarks (e.g., stability and richness) does not affect path integration performance in humans, and indicate that landmark-based navigation does not overshadow path integration (Chen et al., 2017). Similarly, in rodents, navigating with a beacon landmark does not overshadow the ability to return home with self-motion cues after food retrieval (Shettleworth & Sutton, 2005). This mutual independence of landmark-based navigation and path integration is in contrast to the strong competition usually observed between different types of environmental cues (e.g.,Cheng, 1986;Wilson & Alexander, 2008). The relative independence of these two forms of navigation makes it particularly interesting to explore how they are unified to form coherent cognitive maps.

However, it remains uncertain whether landmarks and self-motion cues recruit distinct or common neural processes in the brain. This question has been investigated in non-human animals in the hippocampus (Geva-Sagiv et al., 2016;Markus et al., 1995;Quirk et al., 1990;Radvansky et al., 2021; to name a few) and the retrosplenial cortex (RSC) (Mao et al., 2017,^2020^), but the results have been inconsistent. In contrast, this question has rarely been examined in humans, despite a great number of behavioral studies on the interaction between different spatial cue types in human navigation (e.g.,Newman & McNamara, 2022;Zhao & Warren, 2015). Two notable exceptions are the human fMRI studies conducted byWolbers et al. (2011)andHuffman and Ekstrom (2019), both of which have provided preliminary evidence for cue-independent spatial representations in the human brain.

Recently, we investigated this question with memory-based navigation tasks that dissociate the use of landmarks and visual self-motion cues (i.e., optic flow). We required participants to retrieve memories of the same set of spatial locations using either cue type alone. We found that the right entorhinal cortex (EC) (Chen et al., 2019) and the bilateral RSC (Chen et al., 2024) encoded spatial locations for both landmarks and self-motion cues. Specifically, we observed fMRI adaptation (fMRIa), meaning brain activation changed as a function of spatial proximity between successively visited spatial locations. Furthermore, these fMRIa-based spatial representations were cue specific: in the right EC, different subregions encoded spatial relationships for landmarks and self-motion cues (Chen et al., 2019); in RSC, the voxel-to-voxel pattern of adaptation was distinct between these two cue types (Chen et al., 2024).

Besides the fMRIa analysis, representational similarity analysis (RSA)—a variant of multi-voxel pattern analysis (MVPA)—can also be utilized to examine neural representations. The rationale behind RSA is that the representational similarity between two stimuli, as indexed by the similarity between their multi-voxel activation patterns, is proportional to their similarity, such as in the stimulus space (Kriegeskorte et al., 2008). Here, we speculate that RSA, when combined with the fMRIa analysis, can be leveraged to investigate the cue unification question in spatial navigation for the following two reasons.

First, RSA can potentially reveal neural representations of spatial relationships among locations. While most RSA studies operate on the assumption that neural similarity corresponds to perceptual or conceptual similarities (e.g.,Drucker & Aguirre, 2009;Hatfield et al., 2016), RSA-based coding of spatial distance has been demonstrated in RSC (Peer & Epstein, 2021) and related regions, including the hippocampus (Deuker et al., 2016;Nielson et al., 2015) and occipital place area (Peer & Epstein, 2021). Accordingly, we expect that the RSA approach could capture spatial relationships among locations in regions such as EC and RSC, where closer locations exhibit greater representational similarity. In this sense, the RSA approach shares the basic rationale in assessing neural coding of spatial information with the fMRIa approach, with the key difference being how neural distance between spatial locations is measured (Aguirre, 2007).

Second, RSA can potentially reveal cue-independent spatial representations by transcending lower level physical properties of stimuli. Many studies have shown that the fMRIa and MVPA approaches often yield inconsistent results, leading to the emerging consensus that they interrogate different aspects of the underlying neuronal computations. Specifically, fMRIa-based coding is sensitive to low-level physical properties of stimuli (Epstein et al., 2003;O’Connell et al., 2018;Xu et al., 2007), which resonates with our prior findings that fMRIa-based spatial representations are cue specific in both the right EC and bilateral RSC. On the contrary, MVPA-based coding tends to reflect higher level abstract representations linked to participants’ behavior (Hatfield et al., 2016;Walther et al., 2009;Williams et al., 2007). Accordingly, applying RSA to our existing data might reveal cue-independent spatial representations: RSA-based coding could capture abstract features (i.e., spatial locations) that remain invariant to lower level stimulus properties (i.e., specific spatial cues), aligning with the requirement of retrieving memories for the same locations regardless of the specific cue type present in our tasks (Chen et al., 2019,^2024^).

Given the complementary relationship between the fMRIa and RSA approaches, the simultaneous application of both methods can allow for a more comprehensive understanding of the neural operations underpinning the cue unification process during spatial navigation. Furthermore, when a brain region exhibits both fMRIa- and RSA-based neural representations, a detailed examination of their relationships can provide in-depth insights into the underlying intricate neural computations, thereby going beyond the mere assertion that the brain region houses neural representations for a certain cognitive variable. For example,Mattar et al. (2018)applied both the fMRIa and RSA approaches to investigate object representations in the lateral occipital complex (LOC). LOC exhibited both fMRIa- and RSA-based neural coding for objects, and voxels exhibiting stronger fMRIa-based coding also demonstrated stronger RSA-based coding for objects. These findings support the long-standing hypothesis that neuronal adaptation enhances the representational distinctiveness of different stimuli, thereby improving discrimination performance (Barlow & Földiàgk, 1989).

Here, we aimed to obtain a more complete understanding of how the brain supports the cue unification process during spatial navigation, by investigating RSA effects using the same fMRI datasets that we previously used for the fMRIa analysis. Both of our prior studies contrasted landmarks versus visual self-motion cues in a desktop virtual reality environment. Since the experimental trials were counterbalanced to control for carry-over effects (Aguirre, 2007;Aguirre et al., 2011), we were able to investigate RSA effects in addition to fMRIa effects using the same datasets. To approach a mechanistic understanding of the underlying neural operations, we focused our analyses on the right entorhinal subregions and the bilateral RSC, which had shown fMRIa effects (Chen et al., 2019,^2024^) and may, therefore, be involved in the cue unification process. Comparing neural representations between the two approaches in the same brain regions enabled us to move beyond merely using RSA to detect spatial representations that might have been missed in our previous fMRIa investigations.

To preview, although we did not observe concurrent RSA effects in the right entorhinal subregions, we observed them in RSC for both landmarks and self-motion cues. Furthermore, in RSC, RSA-based spatial representations exhibited opposite properties to those based on fMRIa: while fMRIa-based representations reflected the actual stimulus (i.e., location actually occupied by the participant) and were cue specific (Chen et al., 2024), RSA-based representations reflected the behavior (i.e., location reported by the participant) and were cue independent. Finally, RSA and fMRIa effects were dissociated anatomically, with voxels higher in fMRIa effects not necessarily contributing more to RSA effects than those lower in fMRIa effects. Collectively, our findings demonstrate that RSC contains concurrent cue-specific and cue-independent spatial representations, suggesting that this region plays a critical role in incorporating diverse spatial cues into a unified cognitive map to support navigation behavior.

## 2 Methods

Regarding our first ultra-high field fMRI study (Chen et al., 2019), because we did not observe simultaneous RSA effects in the right entorhinal subregions (Supplementary Information,Section 2.1), for brevity purpose, we do not describe the methods of this study here. Readers can refer to our previous publication.

Regarding our second ultra-high-field fMRI study (Chen et al., 2024), we observed simultaneous RSA effects in the RSC, alongside the previously reported fMRIa effects. Therefore, in the forthcoming paragraphs of this section, we will present a succinct overview of the materials and methods, as these contents have already been described in detail in our previous report.

### 2.1 Participants

Twenty healthy adults from the Magdeburg community participated in this study (10 male; mean age = 25.35 years, standard deviation of age = 3.91 years). All participants were right handed, possessed normal or corrected-to-normal vision, and had no history of neurological diseases. Three additional participants underwent testing but were excluded from data analysis either due to dropping out during the experiment or due to technical problems corrupting the fMRI data. Informed consent was obtained from all participants prior to the experiment, and they received monetary compensation upon completion. The Ethics Committee of the University of Magdeburg approved the experiment.

### 2.2 Stimuli and navigation task

Participants undertook a spatial localization task within virtual environments generated using Worldviz 5.0 (https://www.worldviz.com). Two virtual settings, city and nature (seeFig. 1a), with distinct background views and ground textures were utilized. Both environments featured a linear track covered in the same texture but presented in different colors. The linear tracks shared an identical object configuration, with three arrows and a tree placed along the track layout. The arrows and tree were identical but rendered in different colors in each environment. In between the arrows and the tree were four balls of varying colors placed at evenly spaced locations along the linear track, each separated by intervals of 4 m. To further differentiate between the environments, the order of the four balls was reversed along the track.

**Fig. 1.**
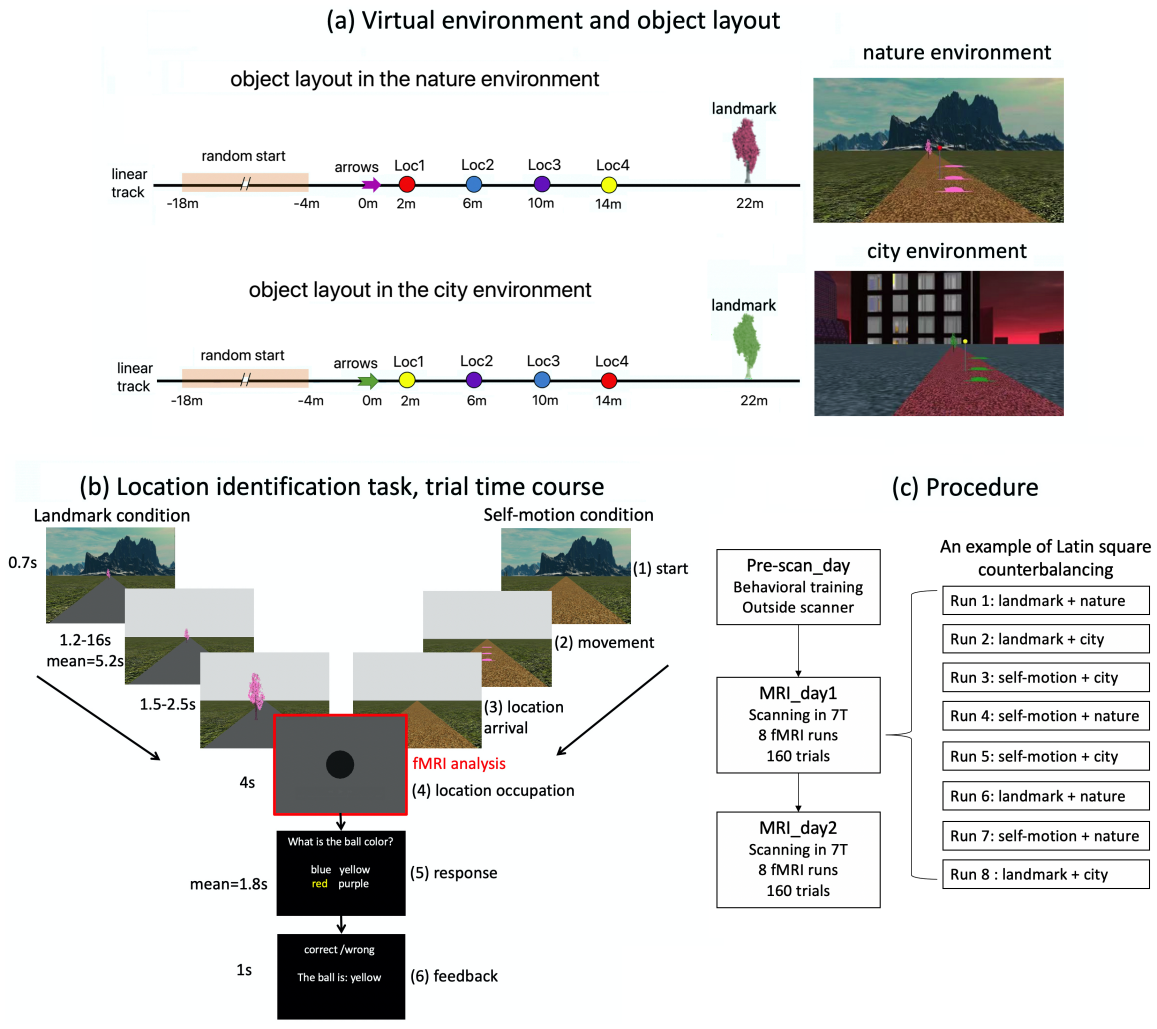
Experimental setup. (a) There were two different virtual environments (left): nature (upper panel) and city (lower panel). The two environments shared the same object layout on the linear track (left). There were arrows, four differently colored balls on poles, and a tree on the linear track. The four balls were positioned at the four test locations, that is, Loc1, Loc2, Loc3, and Loc4. To improve visibility, we used three identical arrows positioned above the ground to denote the same spatial position, meaning that the arrows vertically projected to the same position on the ground and only differed in height. The arrows, the tree, and the floor texture of the linear track had the same physical appearances but in different colors in the two environments. The four balls positioned at the test locations were the same but reversed in order in the two environments. The floor texture outside of the linear track also differed between the two environments. Displayed on the right are snapshots of the two environments, with the background environment, the linear track, the tree, the arrows, and the ball positioned closest to the arrows. (b) The time course of the location identification task. Here, the trial is depicted in the nature environment, which was exactly the same in the city environment. Each trial had six phases. In phase 1 “start,” the participant was positioned at the starting location, which was randomized trial by trial based on a uniform distribution [-18 m, -4 m] (seeFig. 1a, right). In phase 2 “movement,” the participant was passively transported to one of the four test locations. In phase 3, after arriving at the test location, the participant’s first-person perspective was smoothly turned down to vertically face the ground. In phase 4 “location occupation,” the participant’s perspective was fixed at the ground for 4 seconds. In phase 5 “response,” the participant was required to identify the color of the ball positioned at that location within 20 second. In phase 6 “feedback,” feedback was provided, telling the participant whether the response was accurate, and, if incorrect, what the correct answer was. Note that the balls remained invisible throughout the trial, so that participants needed to recall from memory the color of the ball associated with the test location. In the landmark condition, the arrows were invisible, the tree was displayed, and the floor of linear track remained blank. In the self-motion condition, the arrows were displayed, the tree was invisible, and the texture of the linear track was displayed. In both conditions, the background environment only appeared briefly at the beginning of the trial (=0.7 seconds), and disappeared once the passive movement started. The fMRI analyses focused on the 4-second location occupation period (i.e., phase 4), when the visual inputs were the same for both cue conditions (landmark condition & self-motion condition). (c) Participants were familiarized with the virtual environments and trained in the location identification task on the 1^st^day (pre-scan day). On the following 2 days (MRI_day1 & MRI_day2), they completed the location identification task while undergoing MRI scanning in the 7T scanner. In each scanning session, each of the four condition combinations was conducted for two runs, and the eight runs were counterbalanced with the Latin square design, with the restriction that no condition combination occurred consecutively.

#### 2.2.1 Learning task

The purpose of the learning task was to allow participants acquire memories of the four test locations. This task was not accompanied by functional scanning. Participants used a joystick to navigate around in the virtual environments and give responses. Participants were trained to learn four test locations that were evenly spaced on the linear track (Fig. 1a). Four balls of different colors were positioned at the four test locations. Participants needed to remember the colors of the balls associated with the test locations (see the video—the part “LEARNING”).

#### 2.2.2 Test task: Location identification task

Location identification was the focus of the test task. This task was accompanied by functional scanning, which was used to assess neural representations of the four test locations. The trial timeline is illustrated inFigure 1b. In each trial, participants were transported passively to one of the four test locations, stayed for 4 seconds, and were required to recall the color of the invisible ball at that location. The starting position of passive movement was randomly sampled from a uniform distribution ranging from -18 to -4 m on a trial-by-trial basis. This randomization of the starting position dissociated path length and allocentric position of the test location to a certain degree in each cue condition.

The task dissociated self-motion cues and landmark cues, following a similar approach to established behavioral paradigms (Bates & Wolbers, 2014;Chen et al., 2017;Nardini et al., 2008). Cue dissociation was implemented in both the nature and city environments. In the self-motion condition, both arrows and linear track texture were visible, allowing participants to use arrows as an anchor for path integration based on optic flow. Landmarks were not visible to eliminate landmark-based navigation. To prevent association of test locations with isolated ground features, the textures of the linear track and floor outside of it were randomly shifted along the track’s long dimension from trial to trial, following a uniform distribution UD(-50 vm, 50 vm).

Conversely, in the landmark condition, the landmark was visible, enabling participants to rely on it for localization. To eliminate path integration, arrows were invisible, and the ground of the linear track remained blank to remove texture information. Although peripheral optical flow from the floor texture outside of the linear track was present, the randomized starting position of passive movement and the invisibility of the anchoring point for path integration (i.e., arrows) prevented participants from performing path integration to solve the task. This cue manipulation in the landmark condition is akin to the disorientation manipulation typically used to eliminate self-motion information in spatial navigation studies (Cheng, 1986;Sutton et al., 2010).

Note that in the landmark condition, subjects could theoretically use the landmark as a distal anchoring point for path integration. However, because the landmark was located far from the starting position of movement, any anchoring information it provided would be very imprecise, thereby limiting the usefulness of the path integration strategy. This limitation existed even when the floor outside of the track was textured.

In the response phase, the order of the four screen options was randomized across trials, and a randomly chosen option was highlighted as the initial answer. Participants used a specific button on the joystick to loop through options, preventing any fixed associations between test location choices and screen positions or consistent joystick movements. Movement speed was randomly sampled from a uniform distribution ranging from 2 to 5 m/s on a trial-by-trial basis. Accuracy was prioritized but unnecessary delays were discouraged.

### 2.3 Procedure

The experiment took place on 3 separate days, with behavioral training on the 1^st^day (Pre-scan_day) and MRI scanning on the 2^nd^day (MRI_day1) and 3^rd^day (MRI_day2) (Fig. 1c). On the Pre-scan_day, participants went through several cycles of the learning task and the test task to memorize the colors of the balls positioned at the four test locations. Because the Pre-scan day was not our focus of MRI scanning, the associated details are not included here. Readers can refer to our previous report for the details (Chen et al., 2024).

The two scanning sessions, MRI_day1 and MRI_day2, followed the same procedure. Each scanning day began with participants re-familiarizing themselves with the task during structural scanning inside the scanner. This practice lasted approximately 5 minutes and was not subjected to analyses. Subsequently, participants performed the “location identification task” during functional scanning (Fig. 1b). Each scanning day comprised eight runs in total, with two runs for each combination of environment (city vs. nature) and cue condition (self-motion vs. landmark). The eight runs were semi-randomized in order using Latin square designs, with the restriction that the combinations in two consecutive runs must differ within the same day.

We adopted a continuous carry-over design (Aguirre, 2007), using the eight de Bruijn sequences from our previous study with relatively high detection power and low correlation coefficient (Chen et al., 2019). These sequences were generated with 2^nd^order counterbalancing, using the “path-guided” approach (Aguirre et al., 2011). The “carry-over” effect—the influence of a prior item on the brain response to the current item—was counterbalanced, allowing us to rigorously investigate fMRI adaptation and multi-voxel activation pattern similarity simultaneously on the dataset (Aguirre, 2007;Morgan et al., 2011). There were five types of events in each sequence—location occupation periods at the four test locations, in which participants stayed at the test locations for 4s, and null events, in which participants fixated their eyes at a cross displayed in the middle of the blank screen. Each de Bruijn sequence contained 20 location occupation events in total, with 5 repetitions for each location. To allow the hemodynamic response to reach a steady state before the sequence started, we duplicated the very last event in the sequence and placed it at the very beginning. This duplicated event was modeled in the first-level GLMs, but was not included for the analyses of either fMRIa or RSA effects. The eight de Bruijn sequences were randomly assigned to the eight runs in each scanning day for each participant. On each MRI scanning day, the total scanning time lasted up to about 1.75 hours, with the functional scanning up to about 1 hour.

### 2.4 MRI acquisition

Structural and functional images were acquired in a 7T MR scanner (Siemens, Erlangen, Germany) at the Leibniz Institute for Neurobiology in Magdeburg with a 32-channel head coil (Nova Medical, Wilmington, MA). A high-resolution whole-brain T1-weighted structural scan was acquired with the following MP-RAGE sequence: TR = 1700 ms; TE = 2.01 ms; flip angle = 5°; slices = 176; orientation = sagittal; resolution = 1 mm isotropic. A partial-volume turbo spin echo high-resolution T2-weighted structural scan was acquired perpendicular to the long axis of the hippocampus (TR = 8000 ms; TE = 76 ms; flip angle = 60°; slices = 55; slice thickness = 1 mm; distance factor = 10%; in-plane resolution = 0.4×0.4 mm; echo spacing = 15.1 ms, turbo factor = 9, echo trains per slice = 57). Functional scans were acquired with a T2*-weighted 2D echo planar image slab centered on the hippocampus and parallel to its long axis (TR = 2000 ms, TE = 22 ms; flip angle = 85°; slices = 35; resolution = 1 mm isotropic, parallel imaging with grappa factor 1, echo spacing = 0.82 ms). We also obtained 10 volumes of whole-brain functional scans for the purpose of co-registering anatomical masks obtained on the T2-weighted structural scan to functional scans with an MPRAGE sequence (TR = 5000 ms, TE = 22 ms; flip angle = 85°; slices = 100; resolution = 1.6 mm isotropic). The T1-weighted structural image was bias corrected in SPM12. Functional scans were motion and distortion corrected online via point spread function mapping (In & Speck, 2012). Functional scans were left spatially unsmoothed.

**Fig. 2.**
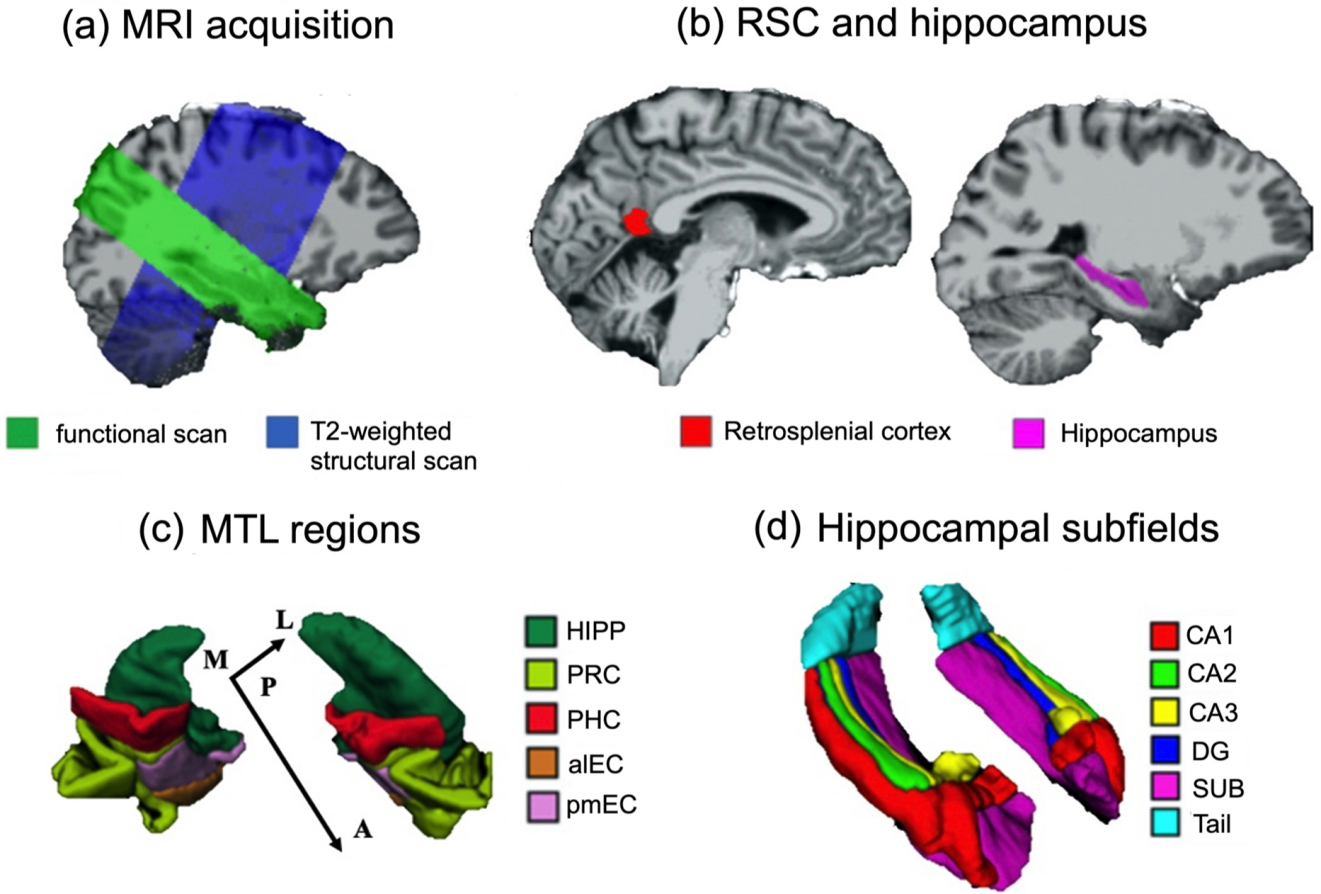
MRI acquisition and anatomical masks of regions of interests. (a) MRI scanning and regions of interest. For an example participant, the functional scan (in green) and the T2-weighted structural scan (in blue) are overlaid on the brain extracted from the T1-weighted structural scan. (b) For an example participant, the anatomical mask of retrosplenial cortex (RSC; in red) and the anatomical mask of hippocampus (HIPP; in violet) are overlaid on the brain extracted from the T1-weighted structural scan. (c) Manually segmented anatomical masks for regions in the medial temporal lobe (MTL) in one example participant. (d) Manually segmented anatomical masks for hippocampal subfields in one example participant. ROI-based RSA was conducted on participants’ native anatomical space (i.e., without normalization or transformation to a standard template). “DG”—dentate gyrus, “SUB”—subiculum, “PRC”—perirhinal cortex, “PHC”—parahippocampal cortex, “alEC”—anterior-lateral entorhinal cortex, “pmEC”—posterior-medial entorhinal cortex. “A”—anterior, “P”—posterior, “M”—medial, “L”—lateral.

Figure 2adisplays the coverage of the functional scan and the T2-weighted structural scan in the T1-weighted structural scan of an example participant’s brain.

### 2.5 Anatomical masks for regions of interest

We obtained anatomical masks of the retrosplenial cortex (RSC) and regions in the medial temporal lobe (MTL) and for each participant in the native brain space.

RSC mask was automatically extracted from each participant’ T1-weighted structural scan (bias corrected in Advanced Normalization Tools (ANTs)) in Freesurfer (Dale et al., 1999). RSC was defined as the posterior-ventral portion of the cingulate gyrus, which mainly consists of BA29/30. Note that this definition of RSC is anatomically different from the retrosplenial complex, which is a functionally defined region typically extending into the parieto-occipital sulcus (Epstein, 2008). For co-registration, the anatomical masks for RSC and its subdivisions were first co-registered to the mean functional scan along with the T1-weighted structural scan in SPM12. Next, the co-registered anatomical masks were resliced using the nearest-neighbor interpolation, with the mean functional scan as the reference image.

The medial temporal lobe consisted of hippocampus, parahippocampal cortex (PHC), perirhinal cortex (PRC), entorhinal cortex (EC). These regions were manually segmented by the author X.C. on the T2-weighted structural scan in ITK-SNAP (Yushkevich et al., 2006;http://www.itksnap.org/pmwiki/pmwiki.php), following the protocol developed by Berron, Vieweg, and colleagues (Berron et al., 2017). The hippocampus was further segmented into different subfields (CA1, CA2, CA3, subiculum (SUB), dentate gyrus (DG), and tail) (Berron et al., 2017). EC was further segmented into the anterior-lateral subregion (alEC) and the posterior-medial subregion (pmEC) on each hemisphere (Chen et al., 2019). The anatomical masks for these MTL regions were co-registered to the mean functional scan of the first scanning day in SPM12, using the same procedure adopted in our previous study (Chen et al., 2019): first, the mean whole-volume functional scan was co-registered to the mean functional scan; second, the T2-weighted structural scan, along with the anatomical masks, was co-registered to the mean whole-volume functional scan obtained from the first step; third, the co-registered anatomical masks were re-sliced using nearest-neighbor interpolation, with the mean functional scan as the reference image.

Figure 2displays all the anatomical masks for our main ROIs in the T1-weighted structural scan and the T2-weighted structural scan of an example participant’s brain.

### 2.6 fMRI analysis

fMRI analyses focused on the location occupation phase, during which the camera faced the blank ground to ensure identical sensory inputs between the landmark and self-motion conditions (Fig. 1b, Phase 4). ROI-based RSA was conducted on participants’ native anatomical space (i.e., without normalization or transformation to a standard template).

**Fig. 3.**
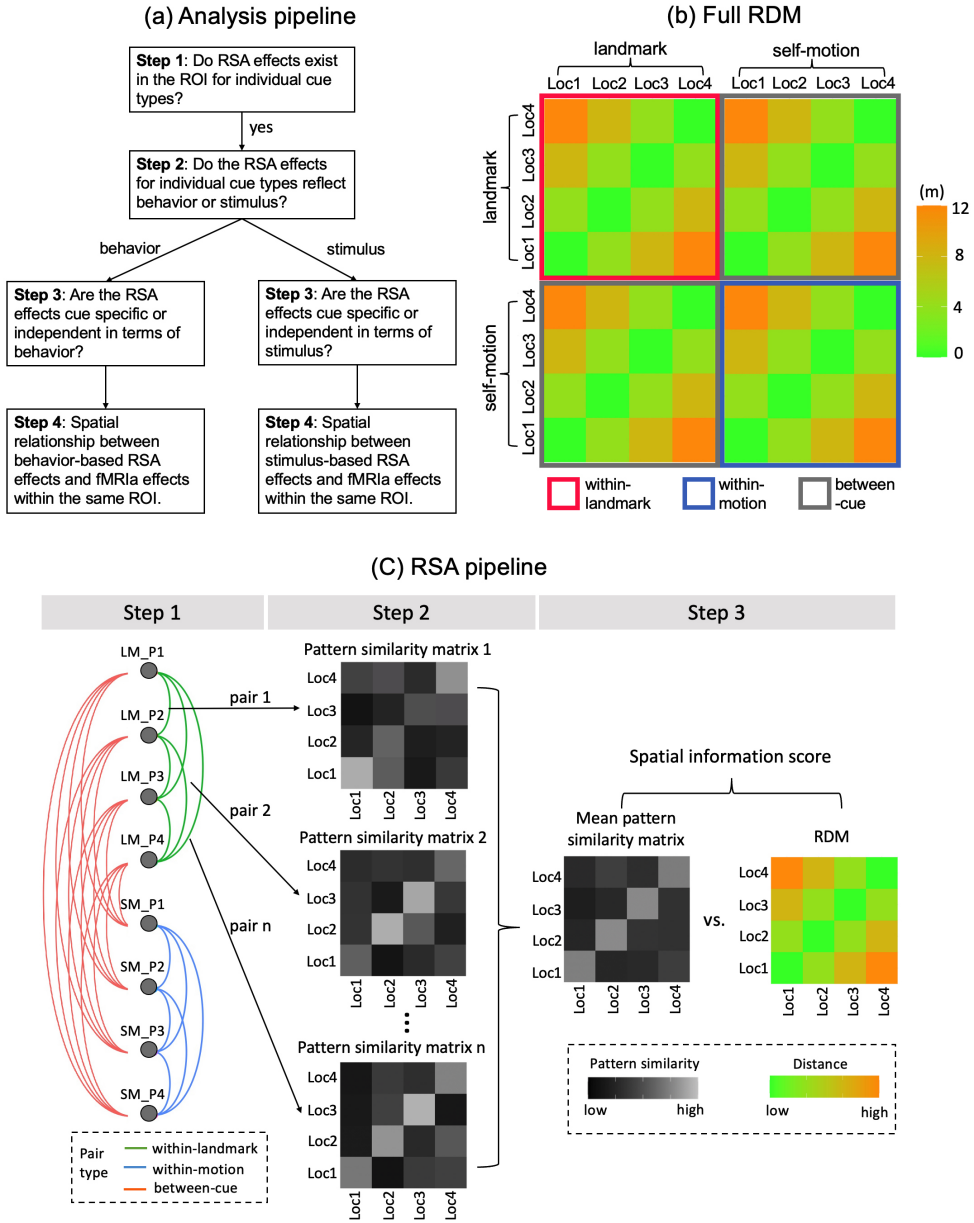
fMRI analysis. (a) Illustrated is the pipeline of our fMRI analysis. We employed the representational similarity analysis (RSA). In Step 1, we examined whether regions of interest (ROIs) exhibited RSA effects for individual cue types separately; that is, we assessed whether the multi-voxel activation pattern similarity between two locations was negatively correlated with the distance between them. In Step 2, we determined whether these RSA effects were associated with stimulus (i.e., location occupied by the participant) or participants’ behavior (i.e., response made by the participant). In Step 3, we analyzed cue specificity/generalizability of the RSA effects. Finally, in Step 4, we investigated the spatial relationship between RSA and fMRIa effects within the same ROI, if both types of effects were present. How Step 3 and Step 4 were conducted depended on the outcome of Step 2. Our analysis particularly focused on brain regions previously identified to show fMRIa effects in our previous reports (Chen et al., 2019,^2024^). (b) Depicted is representational dissimilarity model (RDM), illustrating how representational dissimilarity increases as a function of the spatial relationship between the four test locations. Rows and columns represent the four test locations (Loc1, Loc2, Loc3, Loc4). When the locations are defined by their true locations, the RDM captures location-based positional coding; when defined by participants’ recognition responses, it captures response-based positional coding. The full RDM consists of four sub-RDMs: one capturing RSA effects within the landmark condition (red square), one within the self-motion condition (blue square), and two between landmark and self-motion conditions (two gray squares). (c) Illustrated is the RSA pipeline. In Step 1, LM_P1, LM_P2, LM_P3, LM_P4 stand for the four parts of runs in the landmark condition, with each part containing data from two runs. Similarly, SM_P1, SM_P2, SM_P3, SM_P4 stand for the four parts of the self-motion condition. Activation vectors for test locations were averaged voxel-by-voxel across the two environments in the two runs of each part. In Step 2, the eight parts of the two cue conditions were paired, using the cross-validation approach (Walther et al., 2016). Pairings within the same cue condition (green and blue links) were used to calculate the within-landmark or within-motion spatial information scores. Parings between cue conditions (salmon links) were used to compute the between-cue spatial information score. For each pairing, a 4 x 4 pattern similarity matrix was calculated, denoting the pairwise correlations between activation vectors of test locations. These pattern similarity matrices were then averaged cell-by-cell to produce the mean pattern similarity matrix. In Step 3, the spatial information score was obtained by correlating the mean pattern similarity matrix and the RDM (Fisher transformed and reversed in sign). Values in the matrices are hypothetical data. This illustration depicts the computation of the within-landmark spatial information score. The same logic is applied to the computation of within-motion and between-cue spatial information scores.

Figure 3adepicts the analysis pipeline. First, we adopted the RSA approach to address the question of whether the activity of an ROI encoded allocentric spatial relationships among the four test locations in terms of multi-voxel activation pattern similarity in individual cue conditions (i.e., landmark condition and self-motion condition). The basic rationale is a negative correlation between the distances among test locations and their multi-voxel activation pattern similarities, such that test locations closer to each other showed higher multi-voxel activation pattern similarity.

Second, if spatial coding was detected in an ROI, we assessed whether the spatial coding in individual cue conditions was driven by location (i.e., where the participant was actually located, as indicated by sensory spatial inputs) or response (i.e., where the participants reported they were located, corresponding to the retrieved memory of spatial location). This analysis was motivated by frequent finding of the stronger tie of RSA effects with behavior than stimulus (Bellmund et al., 2019;Hatfield et al., 2016;Koch et al., 2020;Walther et al., 2009;Williams et al., 2007), and our previous finding that fMRIa-based spatial coding in RSC was more strongly tied to stimulus than response (Chen et al., 2024). Assessing the relationship of RSA-based spatial coding with stimulus versus behavior is a critical step in the analysis pipeline, as this assessment would ensure appropriate measurements and adequate statistical power in subsequent analyses.

Third, we assessed whether the RSA-based spatial coding was cue specific or cue independent. How this analysis should be conducted depends on the outcome of the preceding analysis. For example, if the preceding analysis showed that the spatial coding was driven by behavior rather than stimulus, we would assess cue specificity of the spatial coding with respect to behavior instead of stimulus.

Finally, to gain a deeper understanding of the intricate neuronal computations underlying the spatial representations, we evaluated the spatial relationship between the RSA-based spatial coding and the fMRIa-based spatial coding, if both forms of spatial coding existed in the same ROI. Again, the setting of this analysis was also contingent on the outcome of the second analysis, for the purpose of ensuring appropriate measurements and adequate statistical power.

In the following paragraphs, we will describe each step in detail.

#### 2.6.1 Step 1: Analysis of RSA effects for individual cue types

The neural representational similarity between two locations is indexed as the similarity between their multi-voxel activation patterns, which is measured as the correlation between their multi-voxel activation patterns: the higher the correlation, the more similar their neural representations are to each other. Mathematically, the multi-voxel activation pattern is a one-dimensional vector, whose elements represent the activation levels of individual voxels within a brain region. Hence, the size of the multi-voxel activation pattern is equal to the total number of voxels in the brain region. If a brain region contains RSA-based neural representations of spatial relationships among locations, the physical distances between pairwise locations should be negatively correlated with their multi-voxel activation pattern similarities. RSA is a method that belongs to the broader framework of multi-voxel pattern analysis (MVPA).

##### 2.6.1.1 First-level GLM

To capture RSA effects related to location information, we constructed a first-level GLM—*GLM-RSA-location*. This GLM modeled the location occupation phase (Phase 4 inFig. 1b) for the four test locations, using separate regressors. The first location occupation event in each run, which was duplicated to stabilize the hemodynamic response before the sequence started, was modeled with a separate regressor and not analyzed further. To control for potential effects of the preceding movement phase on brain activation during the location occupation phase, we included regressors to model the movement phase (Phase 2 inFig. 1b), independent of test location. Head motion parameters (three rotation parameters and three translation parameters) were included as nuisance regressors. Other phases in the location identification task (Fig. 1b) were not modeled to avoid multicollinearity or due to their brief durations. In particular, since the response phase and the feedback phase were matched among the four test locations, omitting these two phases from the GLM should not affect the spatial coding results from the location occupation phase. For a detailed rationale of the GLM construction, refer to our previous report (Chen et al., 2024).

Nevertheless, to comprehensively assess the impact of the preceding navigation experiences during the location occupation phase, we constructed an additional GLM, which extended the regressors modeling the “movement” phase to encompass the entire navigation stage (Phases 1 + 2 + 3). The RSA results remained consistent with this GLM (seeSection 3.2.7.1“Passive Movement Phase”).

Notably, all first-level GLMs in the present study were constructed in parallel with those used in our previous fMRIa investigation (Chen et al., 2024), ensuring effective comparisons between the RSA and fMRIa effects.

##### 2.6.1.2 Representational similarity analysis (RSA)

After obtaining the beta estimates for the location occupation phase from the first-level*GLM-RSA-location*, we conducted the ROI-based RSA. As illustrated inFigure 3c, The RSA pipeline consists of four steps.

In Step 1, for each cue type and scanning day, the run-specific and location-specific beta estimates for the location occupation phase were divided into two parts chronologically, resulting in four parts in total for each cue type (gray dots inFig. 3c, Step 1). For each cue type, within each part, we averaged out the factor “environment,” which was not of our main interest, by computing the mean activation vector of the two consecutive runs belonging to the two environments for each test location. Each element of the mean activation vector denotes the mean activation level averaged across the two runs of each voxel in the ROI. This resulted in four mean activation vectors for each of the four test locations and each cue type. This approach of averaging out a secondary factor was effectively employed in a previous fMRI study employing the MVPA method (Shine, Valdés-Herrera, et al., 2019). For further justification of this approach, seeSection 3.2.5.

In Step 2, we calculated cross-validated activation pattern similarities, by computing Pearson correlations between the mean activation vectors of pairwise test locations from different parts. This resulted in a 4 x 4 pattern similarity matrix for each part pair, with each cell of the matrix denoting the neural representational similarity between two locations. There were 6 part pairs in total for each within-cue spatial information score, and 16 part pairs in total for the between-cue spatial information score.

In Step 3, the pattern similarity matrices for all part pairs were averaged cell-by-cell, resulting in a 4 x 4 mean pattern similarity matrix. The spatial information score was computed as the Pearson correlation between the Fisher-transformed mean pattern similarity matrix and the RDM. The score was then Fisher transformed and reversed in sign. A positive information score thus indicates that spatial distance information among the test locations is encoded in BOLD signals, meaning that locations are more similar to each other in neural representations as their distance decreases.

Here, we calculated spatial information scores for landmark and self-motion cues separately, meaning that any two mean activation vectors involved in the correlation calculation in step 2 were both from the same cue condition. The spatial information scores were tested using directional one-sample t tests against 0, because we expected closer test locations to exhibit more similar multi-voxel activation patterns. A directional statistical tests are commonly used in studies employing the MVPA approach to examine neural representations of stimuli (Deuker et al., 2013;Morgan et al., 2011;Nielson et al., 2015), as the opposite pattern is difficult to interpret. For comparisons without any specific directional hypotheses, we adopted default tests. For example, when testing the difference between two spatial information scores, we employed a two-tailed t test. For each statistical test, we also calculated the Bayes factor, which indicates the likelihood ratio of the alternative hypothesis over the null hypothesis (BF_10_).

#### 2.6.2 Step 2: Analysis of relationship of RSA effects with stimulus and behavior

We investigated whether RSA effects for individual cue types were tied more strongly to the stimulus received by participants or participants’ behavior. Here, “stimulus” refers to the objective position of the test location defined by the external spatial inputs (i.e., the actual location occupied by the participant). On the contrary, the term “behavior” refers to the response made by the participant (i.e., the location the participant thought herself/himself occupied). When participants made errors, “stimulus” and “behavior” became dissociated.

First, we conducted an RSA-based neural space reconstruction analysis, which recovered the underlying neural space based on pairwise neural representational similarities between locations. We assessed whether the neural space aligned more closely with the stimulus or behavior. Next, we directly contrasted stimulus and behavior in the same first-level GLM, disentangling the contributions of the location-based and response-based distances to neural representational similarity.

##### 2.6.2.1 RSA-based neural space reconstruction

**Fig. 4.**
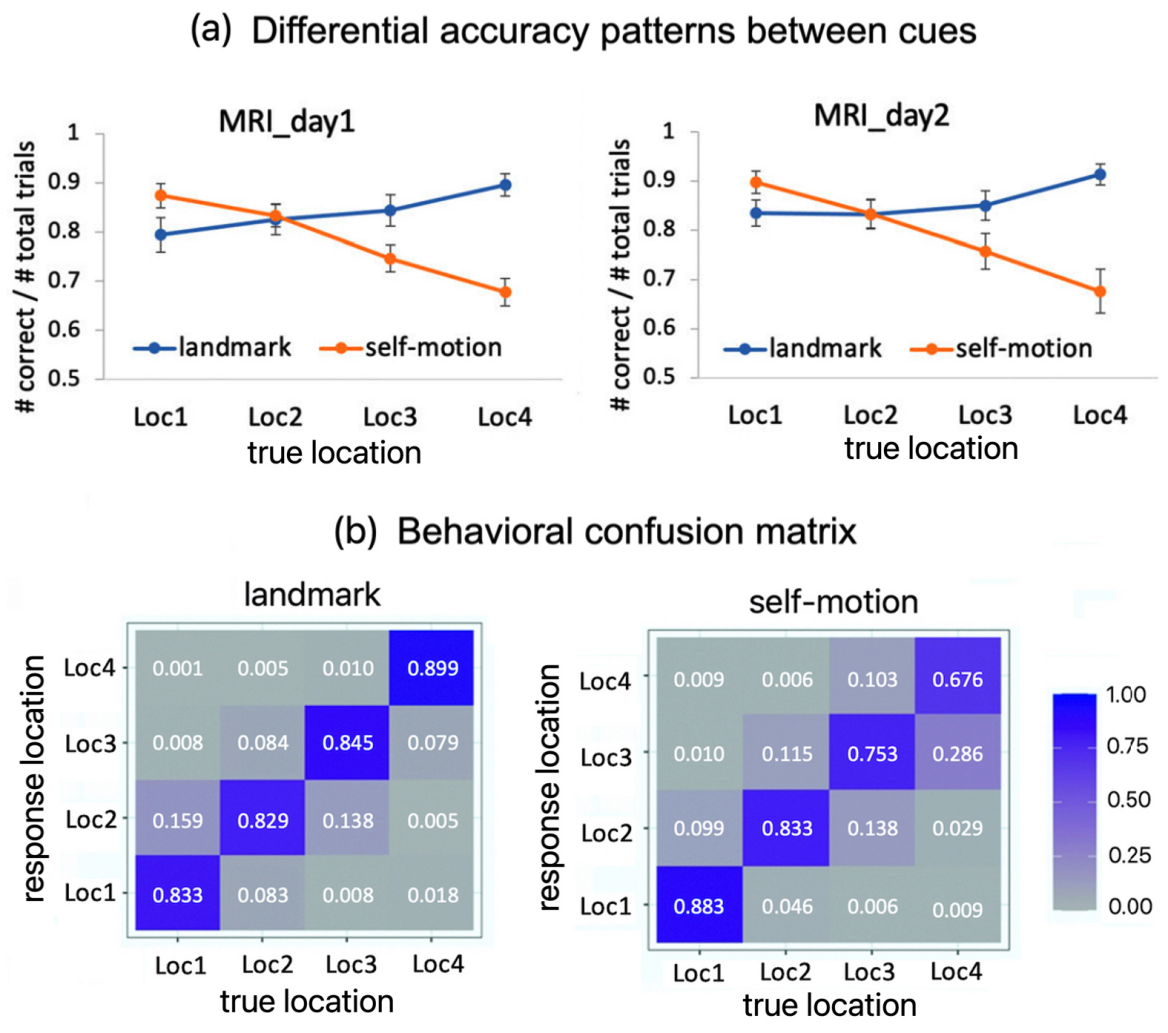
Behavioral findings (Chen et al., 2024). (a) Behavioral accuracy is plotted as a function of cue type and test location on each experimental day. (b) Behavioral confusion matrix for the landmark condition (left) and self-motion condition (right), where columns represent true test locations and rows represent participants’ responses. Cell values denote the proportion of responses. Error bars represent±SE. Adapted from our previous report (Chen et al., 2024).

The basic principle is that locations with more similar multi-voxel activation patterns should be positioned closer to each other in the neural space than those with less similar patterns. In other words, neural representational distinctions determine distances among locations in the neural space. The neural space reconstruction analysis constitutes a deeper analysis of the underlying neural representations, compared with the abovementioned general RSA approach. A major limitation of the general RSA approach is that different location pairs with the same inter-location distance (e.g., Loc1&Loc2 vs. Loc3&Loc4) were treated equally, which could have obscured potential subtle aspects of the underlying neural representations, as suggested by participants’ behavioral performance pattern (Fig. 4). In contrast, the neural space reconstruction analysis overcomes this problem by recovering the entire neural space with positional estimates for all locations.

The neural space reconstruction analysis consisted of three steps (Fig. 6a). In Step 1, we constructed the 4 x 4 neural distance matrix for the four test locations, based on the beta estimates associated with the location occupation phase in*GLM-RSA-location*. Each element of the matrix equaled to 1 minus the Pearson correlation between the multi-voxel activation patterns of two test locations. In this way, elements in the neural distance matrix denote pairwise neural distances among the four test locations. We averaged symmetrical off-diagonal elements in the neural distance matrix. The four diagonal entries were manually set to 0, because multidimensional scaling only exploits relative distances among different items (also seeMarchette et al., 2014). Elements in the matrix were normalized to be within the range [0, 1]: normalized value = (original value – matrix minimum)/(matrix maximum – matrix minimum). In Step 2, multi-dimensional scaling was performed on the normalized neural distance matrix to recover spatial coordinates of the four test locations in the neural space, following the basic principle that locations with greater representational similarities are positioned closer to each other (Kruskal & Wish, 1978). In Step 3, the Procrustes analysis was performed to map the estimated coordinates of locations to the original physical space through rotations and reflections (Gower & Dijksterhuis, 2004).

After obtaining the reconstructed neural space, we evaluated the relationship of location-based RSA effects with behavior versus stimulus. To assess whether the neural space resembled participants’ behavior, we compared the neural space with participants’ behavioral performance. To preview, one of our main behavioral findings was that when participants made mistakes in recalling test locations, most of the time they confused between adjacent locations, for example, reporting Loc2 or Loc4 when occupying Loc3 (Fig. 4b). Mistakes between two locations that were separated by one or more locations (i.e., inter-location distance ≥ 8 m) were extremely rare. Another main behavioral finding is that in the landmark condition, participants were more likely to confuse adjacent locations when they were farther away from the landmark, whereas in the self-motion condition, participants were more likely to confuse adjacent locations when they were farther away from the fixed anchoring point of path integration (Fig. 4a, b). If RSA effects reflected behavior, the underlying neural space should resemble this behavioral pattern by displaying an interaction between cue type and neural distance between adjacent locations along the linear track. To test this possibility, for each cue condition, we quantified neural distance between adjacent locations by computing their distance in the neural space, separately for the three pairs of adjacent locations (Loc1&Loc2, Loc2&Loc3, Loc3&Loc4). The neural distance between adjacent locations was then subjected to a repeated measures ANOVA test, with cue type (landmark vs. self-motion) and adjacent–location pair (Loc1&Loc2 vs. Loc2&Loc3 vs. Loc3&Loc4) as independent variables.

To assess whether the underlying neural space significantly resembled the physical space, we analyzed the group-level neural distance matrix for the sake of maximizing statistical power (Marchette et al., 2014;Persichetti & Dilks, 2019). We adopted a non-parametric permutation approach. First, we obtained the actual Procrustes distance calculated from the group-level neural distance matrix, which indicates the deviation of the reconstructed neural space from the physical space. Second, we applied the permutation procedure to obtain the surrogate distribution of Procrustes distance, to which the actual Procrustes distance would be compared. Specifically, in each permutation, we randomly shuffled the entries in the group-level neural distance matrix. We obtained the Procrustes distance by applying multidimensional scaling and the Procrustes analyses to the shuffled neural distance matrix. This process was repeated 5000 times, resulting in a surrogate distribution of Procrustes distance. Third, the actual Procrustes distance was compared with the surrogate distribution. The significance level (i.e., p value) was calculated as the proportion of values in the surrogate distribution being smaller than the actual Procrustes distance. Significant results (i.e., p < 0.05) would indicate that the group-level neural space resembled the original physical space. This test is inherently one tailed, because a lower Procrustes distance consistently reflects greater similarity between the neural space and the physical space. In other words, deviations of the actual Procrustes distance from the surrogate distribution are expected only in one direction.

##### 2.6.2.2 Direct comparison between stimulus and behavior in RSA effects

We directly contrasted stimulus (i.e., location) and behavior (i.e., response) in RSA effects by estimating their unique contributions to RSA effects for each cue condition (Fig. 7a). Because location and response were dissociated from each other at the trial level (i.e., whether a trial was completed correctly or incorrectly by the participant), we built a new first-level GLM*GLM-RSA-single-trial*, in which individual trials were modeled with separate regressors. Each trial was associated with two labels, location and response. Whether the two labels matched depended on whether the participant completed the trial correctly by retrieving the correct memory for the occupied location.

Next, we computed cross-validated Pearson r correlation between single-trial-based multi-voxel activation patterns from pairwise runs, resulting in a 20 x 20 activation pattern similarity matrix for each run pair in the neural space, as there were 20 effective trials in each run (Fig. 7a, the gray matrix). In addition, two 20 x 20 distance matrices were constructed, one based on location and the other on response (Fig. 7a, the two green–orange matrices). When based on location/response, the distance matrix denotes pairwise objective/subjective distances between test locations. For example, if the participant reported Loc2 while occupying Loc1 in one trial and reported Loc4 while occupying Loc2 in another trial, the distance between the two trials was 8 m in terms of response and 4 m in terms of location.

Next, we used these two distance matrices (standardized) to jointly predict the activation pattern similarity matrix (Fisher transformed and standardized), using the multiple linear regression analysis. The two estimated regression coefficients (i.e., beta-unique, reversed in sign) denoted the respective unique contributions of the two predictors, with the contribution of the other predictor excluded. The multiple linear regression was performed for each run pair, and the estimated regression coefficients were averaged across all run pairs to obtain the final estimates. These estimates were tested against 0 using directional one-sample t tests, with Bayes factors (BF_10_) computed.

This analysis was conducted for the landmark condition and the self-motion condition separately, meaning that the two runs in each run pair were from the same cue condition.

#### 2.6.3 Step 3: Analysis of cue specificity/generalizability of RSA effects

After determining whether RSA-based spatial representations were driven by stimulus or behavior, we evaluated whether these representations were cue specific or cue independent. If stimulus was the driving factor, cue specificity was evaluated based on the true locations using the beta estimates from the location occupation phase in the previously described first-level GLM (*GLM-RSA-location*). In this GLM, within each run, the location occupation events were modeled with the same event regressor, irrespective of the test location. In contrast, if behavior was the driving factor, the analysis was conducted based on participants’ responses. A new first-level GLM,*RSA-GLM-response*, was constructed, which had the same structure as*RSA-GLM-location*, except that location occupation events were classified by the participant’s responses rather than the actual occupied locations. In each run, events with the same response were modeled with the same event regressor.

Cue specificity of spatial representations was assessed by calculating the between-cue spatial information score. The calculation of this score was similar to the previously mentioned within-cue spatial information score (Section 2.6.1), with one key difference. In the within-cue calculation, any two multi-voxel activation patterns associated with two locations in the correlation calculation were from the same cue condition. In contrast, in the between-cue calculation, the two multi-voxel activation patterns associated with two locations were from different cue conditions (seeFig. 3c, Step 1). A significantly greater than 0 between-cue spatial information score indicates that spatial representations are generalizable between different cue types; otherwise, cue-specific representations are implied.

#### 2.6.4 Step 4: Spatial relationship between RSA and fMRIa effects

To assess the spatial relationship between fMRIa and RSA effects, we first ranked voxels in the ROI by the magnitude of landmark or self-motion fMRIa effect (signed and spatially unsmoothed) from low to high. The ranked voxels were then divided into four equally sized quarters. We compared spatial information scores for the four quarters (Mattar et al., 2018).

Additionally, we compared quarter-wise spatial information scores with empirical chance levels. To calculate these levels, we randomized the order of the voxels, rather than ranking them by adaptation magnitude. For each randomization, we calculated the spatial information scores for each quarter of voxels. Voxel randomization was performed for 1000 times, and the mean spatial information scores were averaged across all the randomizations, which was taken as the empirical chance levels.

Similar to the previously described assessment of cue specificity/generalizability of spatial representations, RSA measurements here should depend on the outcome of the comparison between location and response. If stimulus was the driving factor behind the RSA effects, then the RSA measurements should be defined by true positions of the test locations. Conversely, if the response was the driving factor, then the RSA measurements should be defined by participants’ responses.

Notably, all RSA-related analyses in the present study were conducted in parallel with our previous fMRIa analyses (Chen et al., 2019,^2024^), ensuring effective comparisons between the RSA and fMRIa effects. This methodological parallelism arises from the conceptual similarity between the RSA and fMRIa analyses. The key difference between the RSA and fMRIa approaches lies in how neural representational similarity is measured. In RSA, neural representational similarity between two locations is indexed by their multi-voxel activation pattern similarity, whereas in the fMRIa analysis, it is indexed by the brain activation level for one location when preceded by the other location.

## 3 Results

To reiterate, we had a particular focus on brain regions that already showed fMRIa-based spatial coding in our previous reports, specifically the right EC subregions in our first fMRI study (Chen et al., 2019) and RSC in our second fMRI study (Chen et al., 2024). To preview, we did not observe concurrent RSA effects in the right EC subregions in the first study (Chen et al., 2019;Supplementary Information,Section 2.1). However, in the second study (Chen et al., 2024), we observed concurrent RSA effects in RSC. Therefore, here we report and discuss the results from the second study.

### 3.1. Previous behavioral results

The behavioral results have been described in detail in our previous report (Chen et al., 2024), and hence we only summarize key findings here (Fig. 4). Participants showed distinct behavioral accuracy profiles across the test locations between the landmark condition and the self-motion condition (Fig. 4a), such that in the landmark condition, accuracy increased as the test location became closer to the landmark, while in the self-motion condition, accuracy increased as the test location became closer to the fixed starting point of path integration (interaction between cue type and the linear trend of test location, t(57) = 8.487; p < 0.001).

We also presented the behavioral confusion matrix, which showed more details of participants’ behavior (Fig. 4b). In both cue conditions, when participants made mistakes, they often confused the target location with its adjacent locations, for example, confusing Loc1 with Loc2. The confusion between two locations that were more than one location apart was very rare, for example, confusing Loc1 with Loc3. Together, the behavioral results implied that our manipulation of cue dissociation was successful.

### 3.2 Current fMRI results from representational similarity analysis (RSA)

We re-analyzed the fMRI dataset from our previous report (Chen et al., 2024), using the RSA approach. As described in the Methods section, the current fMRI analyses adhered to the following pipeline (Fig. 3): First, we tested for RSA effects reflecting the coding of spatial relationships between test locations, separately for the two cue types. Next, we examined the nature of the RSA effects by assessing whether these effects were driven by stimulus or behavior. After that, we evaluated whether the RSA effects were cue specific or cue independent. Finally, we compared the RSA effects with our previously reported fMRIa effects. The third step and the fourth step are contingent on the outcome of the second step.

#### 3.2.1 RSC showed location-based RSA effects for both cue types

To test whether RSC contained neural coding of spatial relationships between test locations for individual cue types, we calculated a*spatial information score*, quantified as the correlation between inter-location distances and multi-voxel activation pattern dissimilarities (see Methods,Section 2.6.1.2for detailed methodology).

**Fig. 5.**
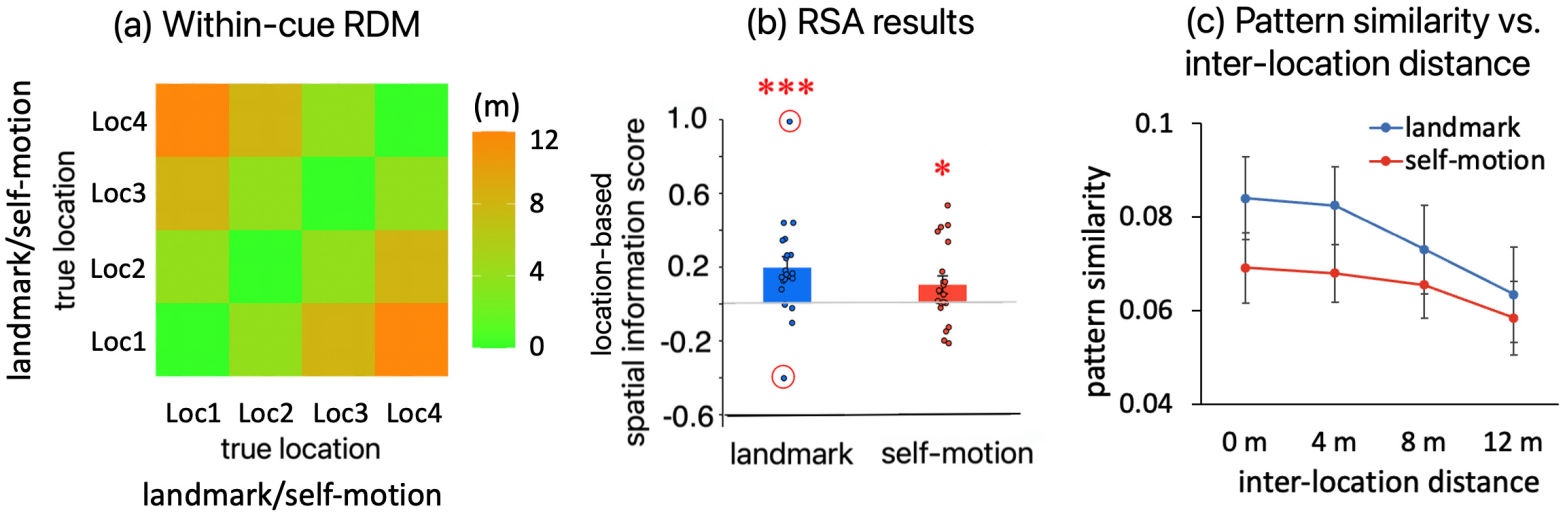
Location-based RSA effects for individual cue types in RSC. (a) The RDM for calculating location-based within-cue RSA effects. This RDM contains physical distances between pairwise locations from the same cue condition. This means that trials were categorized according to the actual locations occupied by the participant. The location-based spatial information scores were calculated based on this RDM. (b) RSA results. Location-based spatial information score is plotted as a function of cue type (landmark vs. self motion). Inside the red circles are statistical outliers, which are greater than t interquartile ranges above the 3^rd^quartile or less than 3 interquartile ranges below the 1^st^quartile. “*”—p_1-tailed_< 0.05, “**”—p_1-tailed_< 0.01, “***”—p_1-tailed_< 0.001. When testing whether the spatial information score was greater than 0, one-tailed t test was adopted. (c) To visualize the significant RSA effects shown in (b), activation pattern similarity is plotted as a function of inter-location distance defined by true test locations for each cue type. Error bars represent±SE.

As shown inFigure 5b(left panel), in RSC, spatial information score was significantly greater than 0 in both the landmark condition (t(19) = 3.207, p_1-tailed_= 0.002, BF_10_= 19.486; two outliers winsorized, t(19) = 4.822, p_1-tailed_< 0.001, BF_10_= 478) and the self-motion condition (t(19) = 1.938, p_1-tailed_= 0.034, BF_10_= 2.109). To visualize the significant spatial information scores, the multi-voxel pattern similarity decreased gradually as inter-location distance increased (Fig. 5b, right panel).

These findings converge with our previous finding of fMRIa-based spatial coding for both cue types in RSC (Chen et al., 2024), implying that this structure plays a crucial role in processing spatial information stemming from diverse sensory sources.

#### 3.2.2 RSA-based spatial coding in RSC reflects behavior rather than stimulus

Here, we sought to compare the roles of stimulus and behavior in the RSA effects in RSC. This analysis was motivated by the frequent finding that MVPA effects in general are strongly linked to participants’ overt behavior (Hatfield et al., 2016;Vass & Epstein, 2013;Walther et al., 2009;Williams et al., 2007) and our previous observation that the fMRIa effects were more strongly associated with stimulus than behavior (Chen et al., 2024). Determining the driving factor behind this coding is crucial for adequately assessing its cue specificity/generalizability in the subsequent analysis—the core question we aimed to answer.

##### 3.2.2.1 RSA-based neural space reconstruction

The neural space reconstruction analysis aimed to recover the entire neural space with positional estimates for all four test locations, based on the rationale that locations with more similar multi-voxel activation patterns should be positioned closer to each other in the neural space (see Methods,Section 2.6.2.1for detailed methodology).

First, we assessed whether the neural space resembled participants’ behavior by comparing the neural space with participants’ behavioral performance pattern, as depicted inFigure 4(see Methods,Section 2.6.2.1for detailed methodology; also seeFig. 6a). To reiterate, after obtaining participant-specific neural spaces, for each cue condition, we quantified neural distance between adjacent locations by computing their distance in the neural space, separately for the three pairs of adjacent locations (Loc1&Loc2, Loc2&Loc3, Loc3&Loc4). The neural distance between adjacent locations was then subjected to a repeated measures ANOVA test, with cue type (landmark vs. self-motion) and adjacent–location pair (Loc1&Loc2 vs. Loc2&Loc3 vs. Loc3&Loc4) as independent variables. As shown inFigure 6b.1, we observed a significant interaction between cue type and the linear trend of adjacent–location pair (F(1, 19) = 12.016, p = 0.003,$\eta_{\text{p}}^{2}$= 0.387): in the landmark condition, the neural distance between adjacent locations decreased as the locations got farther away from the landmark, whereas the opposite occurred in the self-motion condition. Because shorter neural distance means more similar neural representations, this finding is parallel to the behavioral finding that in the landmark condition, locations farther away from the landmark were behaviorally more confusable than those closer to the landmark, whereas the opposite occurred in the self-motion condition (Fig. 4).

**Fig. 6.**
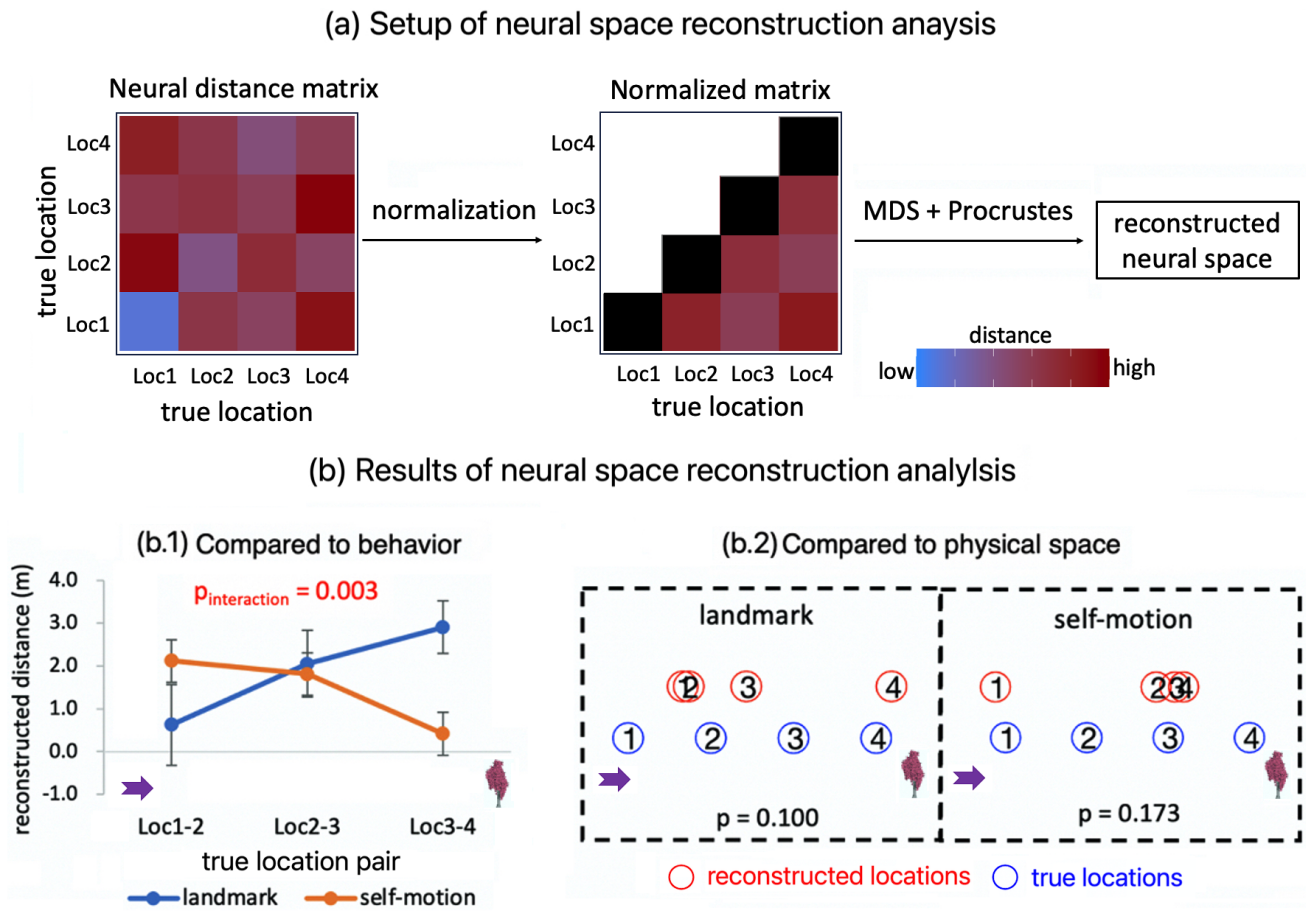
RSA-based neural space reconstruction in RSC. (a) Setup of the analysis. First, a 4 x 4 neural distance matrix was constructed, with the elements denoting pairwise neural distances among the four test locations. Neural distance was measured as 1—pattern similarity. Next, the neural distance matrix was normalized, so all the elements were within the range [0, 1]. The four on-diagonal elements were manually set to 0 (dark cells), as this analysis only exploits neural dissimilarities between different items. The normalized neural distance matrix was then subjected to the multi-dimensional scaling analysis, followed by the Procrustes analysis to reconstruct the neural space. Here, the matrices contain hypothetical data. (b) Results of the analysis. In the left panel (b.1), the neural space was compared with participants’ behavior. The reconstructed distance between adjacent locations is plotted as a function of location pair and cue type. The interaction between the linear trend of location pair and cue type was significant (p = 0.003), resembling the behavioral performance pattern as shown inFigure 4. In the right panel (b.2), the group-level neural space (red circles) was compared with the physical space (blue circles), via a permutation test. Numbers in the circles denote the four test locations (e.g., “1” indicates Loc1). The group-level neural space did not significantly resemble the physical space for either cue type (p’s≥0.1). Error bars represent±SE. The arrow and the tree are displayed alongside the results to indicate the test locations’ positions relative to the anchoring points for path integration in the self-motion condition and for landmark-based navigation in the landmark condition.

Next, we assessed whether the underlying neural space significantly resembled the physical space. To reiterate, we compared the actual Procrustes distance with the surrogate distribution obtained by permuting cells in the group-level neural distance matrix (see Methods,Section 2.6.2.1for detailed methodology). As shown inFigure 6b.2, the group-level neural space did not significantly resemble the physical space in either the landmark condition (p = 0.102) or the self-motion condition (p = 0.158). Furthermore, the group-level neural space exhibited a structure qualitatively similar to the behavioral performance pattern in both cue conditions (Fig. 4); that is, the neural distance was larger for locations closer to the landmark in the landmark condition, and the opposite pattern occurred in the self-motion condition.

In brief, in RSC, the RSA-based neural space did not resemble the physical space, indicating that the physical space was not represented faithfully in this region. In contrast, the neural space exhibited a pattern parallel to that of participants’ behavioral performance, suggesting that the deviations of the RSA-based neural representations from the physical space might have mediated the mistakes participants made in their behavior.

##### 3.2.2.2 Direct comparison between stimulus and behavior in RSA effects

**Fig. 7.**
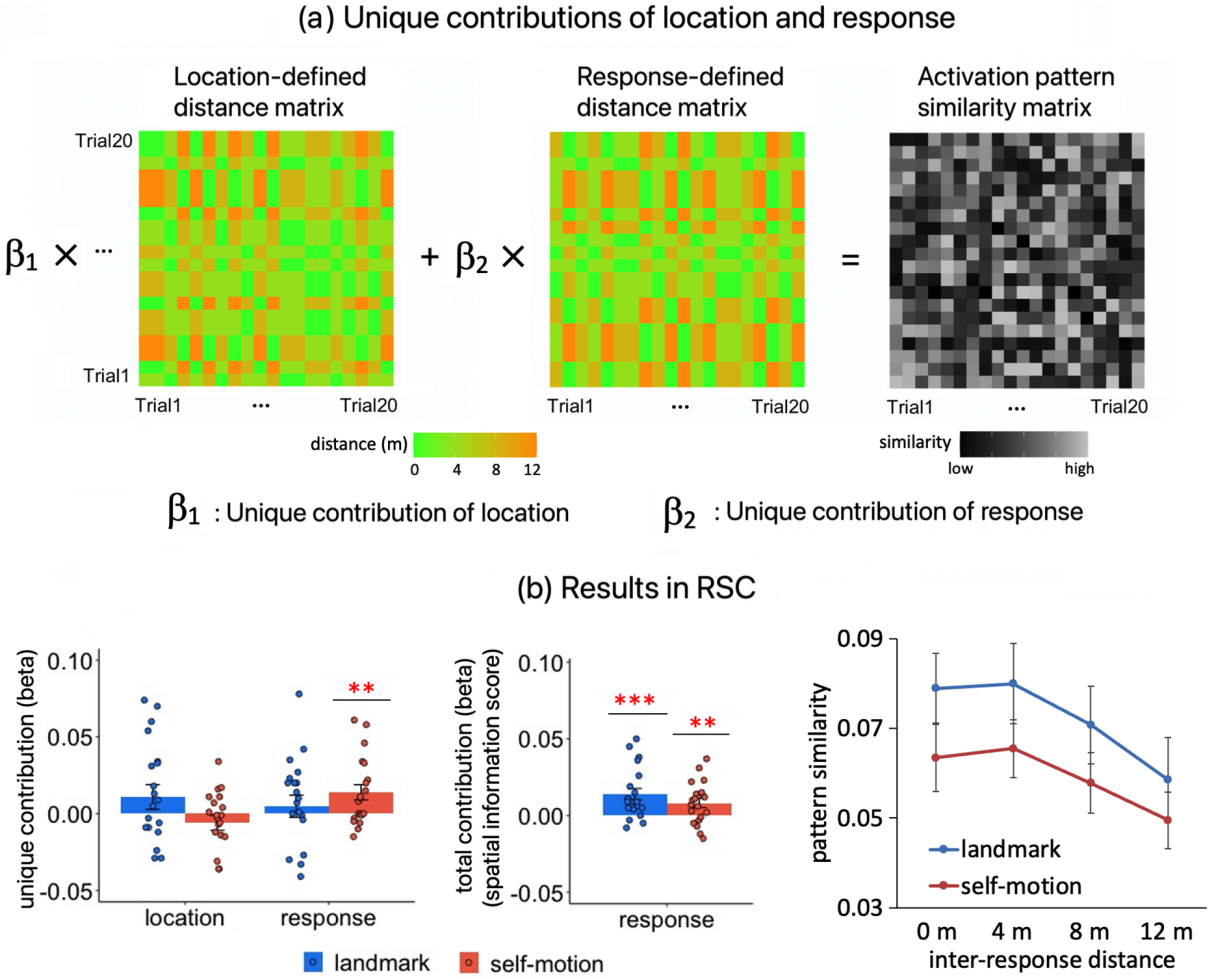
Direct comparison between location and response in RSA effects in RSC. (a) We disentangled location and response by estimating their unique contributions to RSA effects. Each run contained 20 trials in a given cue condition. For each run pair, we calculated three measurements for each trial pair: the location distance based on the true positions of the test locations, the response distance based on participants’ responses (i.e., retrieved location memories), and the multi-voxel activation pattern similarity, resulting in three 20 x 20 matrices. For each run pair, we conducted a multiple linear regression with the two distance matrices as the independent variables and the multi-voxel activation pattern similarity matrix as the dependent variable. The estimated beta coefficients ($\beta_{1}$and$\beta_{2}$) were then averaged across all pair runs, which were taken as the unique contributions of location and response to RSA effects. Here, all matrices contain hypothetical data. (b) The left panel displays the unique contributions of location and response in each cue condition. This analysis also allowed us to estimate the total contribution of response to RSA effects, that is, response-based RSA effects, when the other independent variable was not included in the multiple linear regression (a.3). Results for response-based spatial information scores are displayed in the middle panel. The right panel visualizes the response-based RSA effects as displayed in the middle panel, by plotting multi-voxel activation pattern similarity as a function of inter-response distance for each cue condition. “*”—p_1-tailed_< 0.05, “**”—p_1-tailed_< 0.01, “***”—p_1-tailed_< 0.001. When testing whether the spatial information score was greater than 0, the one-tailed t test was adopted, because we had a specific directional hypothesis that pattern similarity should increase as inter-response/inter-location distance decreased. Error bars represent±SE.

To compare stimulus and response directly, we estimated their unique contributions to the RSA effects while accounting for each other. We used location- and response-defined distances among individual location occupation events as independent variables to jointly predict multi-voxel activation pattern similarity between locations (Fig. 7a; also see Methods,Section 2.6.2.2for detailed methodology).

As shown inFigure 7b(left panel), in the self-motion condition, the unique contribution of location was not significant and was even negative numerically (t(19) = -1.274, p_1-tailed_= 0.866, BF_10_= 0.114), whereas the unique contribution of response was significant (t(19) = 2.806, p_1-tailed_= 0.004, BF_10_= 9.198). This result indicates that RSA-based spatial coding for self-motion cues was driven by response rather than location. In the landmark condition, however, neither the unique contribution of location nor response was significant (location, t(19) = 1.343, p_1-tailed_= 0.099, BF_10_= 0.904; response, t(19) = 0.674, p_1-tailed_= 0.258, BF_10_= 0.419). This result suggests that in the landmark condition, location and response could not be dissociated, probably due to the very high behavioral accuracy, which caused a high correlation between the two independent variables.

One potential limitation of this control analysis is the high behavioral accuracy exhibited by our participants in the landmark condition (mean ACC = 0.851), as dissociation between objective location and subjective response depends on the number of errors made by participants. On the contrary, behavioral accuracy was significantly lower in the self-motion condition (mean ACC = 0.851 vs. 0.786, F(1, 19) = 10.552; p = 0.004,$\eta_{\text{p}}^{2}$= 0.357), ensuring the reliability of the stimulus–behavior dissociation results in this condition.^*^As the results are less conclusive for the landmark condition, future studies employing more challenging tasks are needed to examine stimulus–behavior dissociation in RSA-based spatial coding during landmark-based navigation.

Taken together, these findings indicate a stronger relationship between the RSA effects and behavior compared with stimulus, though these two factors could not be unequivocally dissociated in the landmark condition due to the high behavioral accuracy in this condition.

##### 3.2.2.3 Summary

The neural space reconstruction analysis and unique contribution analysis yielded relatively convergent results: RSA-based positional coding in RSC was more strongly tied to behavior than to stimulus. This finding contrasts sharply with our previous finding that fMRIa-based positional coding in RSC was more strongly linked to stimulus than to behavior (Chen et al., 2024).

Since the positional coding in RSC was mainly driven by response rather than location, we observed significant response-based spatial information score in each cue condition, which corresponds to the total contribution of response to multi-voxel activation pattern similarity between locations (landmark, t(19) = 3.769, p_1-tailed_< 0.001, BF_10_= 58.228; self-motion, t(19) = 2.744, p_1-tailed_= 0.006, BF_10_= 8.212;Fig. 7b, middle panel). To visualize these effects, the multi-voxel pattern similarity decreased gradually as response-defined distance between location occupation events increased (Fig. 7b, right panel).

#### 3.2.3 RSA-based spatial coding in RSC is cue independent in terms of response

Given our preceding finding that in RSC, the RSA-based spatial coding in individual cue conditions was more strongly tied to behavioral output (response) than stimulus input (location), we assessed whether this coding was cue specific or cue independent in terms of response. By selecting the driving factor—response instead of location—to model the RSA effects, we ensured that our measurement adequately captured the nature of the underlying neural code.

First, to calculate response-based spatial information score, we constructed a new first-level GLM (*RSA-GLM-response*) with the same structure as the original first-level GLM for location-based RSA effects (*RSA-GLM-location*). The only difference is that in*RSA-GLM-response*, the location occupation event was defined and grouped by the participant’ response instead of the actual location occupied by the participant. Next, we assessed cue specificity of the coding by computing the response-based between-cue spatial information score. This computation was similar to the within-cue spatial information scores but involved correlating multi-voxel activation patterns from different cue conditions instead of from the same cue condition. We correlated the between-cue multi-voxel activation pattern similarity with response-defined distance, which was then Fisher transformed and reversed in sign. If the response-based between-cue spatial information score is significantly greater than 0, it indicates that the positional coding was generalizable between cue types (see Methods,Section 2.6.3for detailed methodology).

**Fig. 8.**
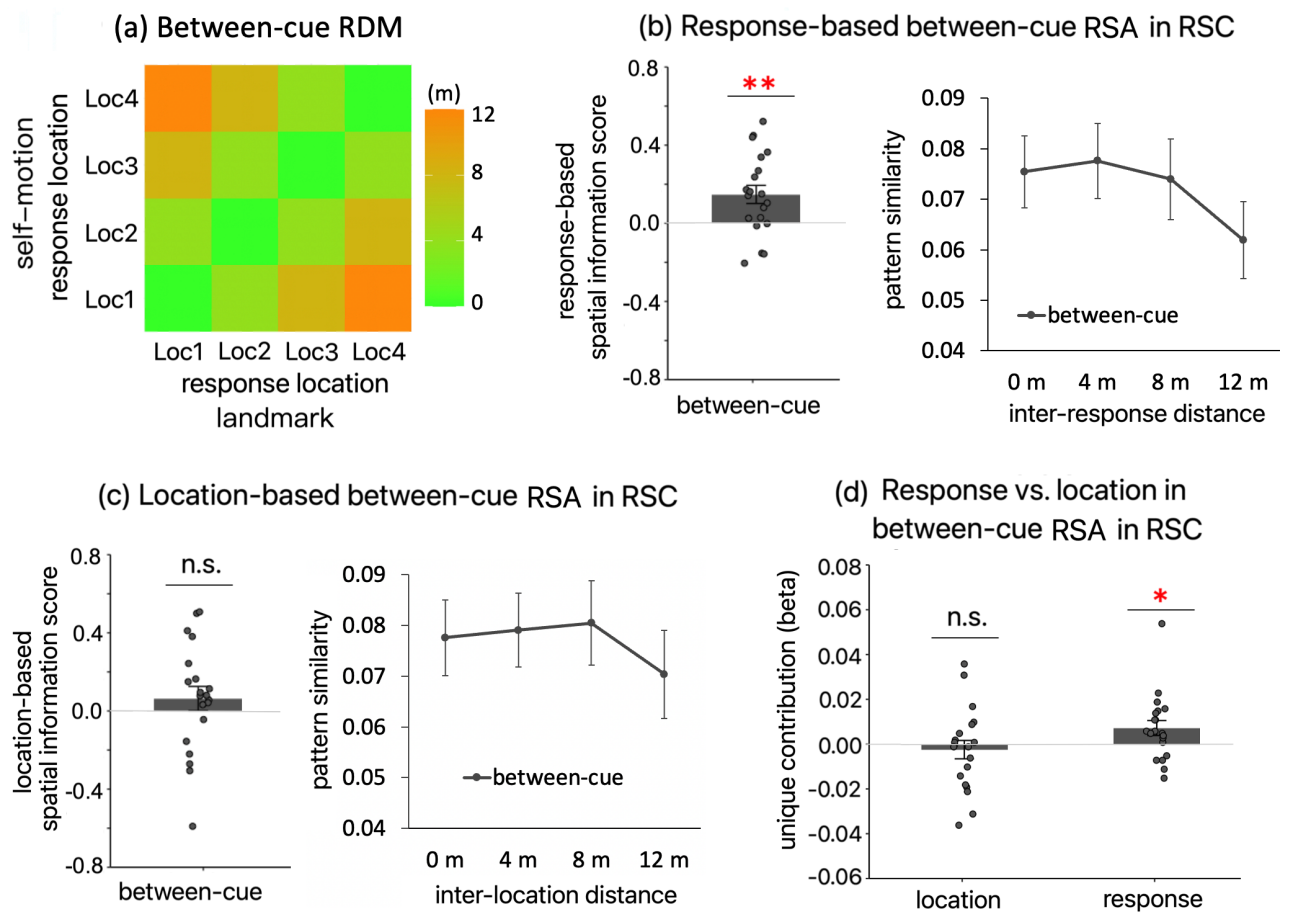
Response-based between-cue RSA effect in RSC. (a) The RDM used for calculating the response-based between-cue RSA effect. Cells of this RDM contain response-defined distances between pairwise responses from different cue conditions. Trials were categorized according to participants’ responses in the first-level GLM in the first place. (b) The left panel displays the response-based between-cue RSA effect. The right panel visualizes this significant effect by plotting between-cue multi-voxel activation pattern similarity as a function of response distance. (c) For completeness, we also calculated the location-based between-cue RSA effect, with the location occupation events defined and grouped by true positions of the test locations in the first-level GLM. This effect was not significant. (d) Unique contributions of location and response to the between-cue RSA effect, following the procedure illustrated inFigure 7a. “*”—p_1-tailed_< 0.05, “**”—p_1-tailed_< 0.01, “***”—p_1-tailed_< 0.001. When testing whether the spatial information score was greater than 0, one-tailed t test was adopted. Error bars represent±SE.

As shown inFigure 8b(left panel), in RSC, the response-based between-cue spatial information score was significant (t(19) = 3.143, p_1-tailed_= 0.003), with a Bayes factor indicating strong evidence for the alternative hypothesis (BF_10_= 17.248 > 10). A visualization of this effect shows that as response-defined distance increased, between-cue multi-voxel pattern similarity gradually decreased (Fig. 8b, right panel).

Furthermore, we found that the between-cue spatial information score was driven by response rather than location, same as the within-cue spatial information scores. First, we computed the location-based between-cue spatial information score using the original GLM assessing location-based RSA effects (*GLM-RSA-location*). This score was not significant (t(19) = 1.014, p_1-tailed_= 0.162, BF_10_= 0.604;Fig. 8c). Next, we disentangled location and response in explaining the between-cue spatial information score by calculating their unique contributions, using the same method as for the within-cue spatial information scores (Fig. 7a). The unique contribution of response was significant (t(19) = 2.220, p_1-tailed_= 0.017, BF_10_= 3.313), while the unique contribution of location was not significant and numerically negative (t(19) = -0.546, p_1-tailed_= 0.667, BF_10_= 0.162;Fig. 8d). These results suggest, again, that the RSA-based positional coding in RSC was more strongly linked to participants’ behavior than to the actual stimulus.

To further characterize the response-based RSA effects in RSC, we subjected multi-voxel pattern similarity to a repeated-measures ANOVA, with measurement type (within landmark vs. within motion vs. between cue) and inter-response distance (0 m vs. 4 m vs. 8 m vs. 12 m) as independent variables. As expected, the main effect of inter-response distance was significant (F(3, 57) = 18.563, p < 0.001,$\eta_{\text{p}}^{2}$= 0.494). We then conducted the trend analysis. The linear trend was significant (t(57) = -6.621, p < 0.001), so was the quadratic trend (t(57) = -3.399, p = 0.001). The cubic trend was not significant (t(57) = 0.549, p = 0.585). These results suggest that the relationship between multi-voxel pattern similarity and inter-response distance was not strictly linear. Additional analyses showed that this nonlinear relationship was primarily driven by the fact that multi-voxel pattern similarity decreased as inter-response increased only when different test locations were compared: multi-voxel pattern similarity did not differ between 0 m and 4 m (t(57) = 0.657, p = 0.514), was lower at 8 m than 4 m (t(57) = -2.615, p = 0.011), and was lower at 12 m than at 8 m (t(57) = -4.150, p < 0.001).

In summary, our results showed that in RSC, the RSA-based spatial coding, which primarily reflected response, was generalizable between cue types. However, this spatial coding was not Euclidean, as the relationship between multi-voxel pattern similarity and inter-response distance was not strictly linear. This nonlinear relationship aligns with the frequent behavioral finding that spatial representations do not reflect the true Euclidean spatial relationships among locations (Warren et al., 2017). However, the interpretation of this nonlinear relationship is complicated by the nonlinear relationship between stimulus strength and neuronal activity (Yang et al., 2021) and the elusive relationship between neuronal activity and fMRI BOLD signals (Logothetis & Wandell, 2004).

#### 3.2.4 Robustness of response-based spatial coding in RSC

In retrospect, in the ROI-based analysis of representational similarity for RSC, we tested the statistical significance of six spatial information scores using six one-sample t tests in total—within landmark, within motion, and between cue, based on location or response. This approach involves multiple comparisons, which can result in false positives. To control for the familywise Type I error, we corrected for multiple comparisons across all six one-sample t tests using a permutation-based Max-T test, which is analogous to the Holm–Bonferroni approach (Supplementary Information,Section 1). To ensure a fair comparison between location and response, we recalculated the response-based within-cue spatial information scores using*GLM-RSA-response*. These recalculated scores were very close to those obtained using*GLM-RSA-single-trial*.

The three response-based spatial information scores (using*GLM-RSA-response*) and the three location-based spatial information scores (using*GLM-RSA-location*) were subjected to the multiple comparisons correction. All three response-based scores remained significant: within landmark, p_corrected_= 0.004; within motion, p_corrected_= 0.049; between cue, p_corrected_= 0.009. In contrast, the location-based scores remained significant in the landmark condition (p_corrected_= 0.011), but not in the self-motion condition (p_corrected_= 0.068). The location-based between-cue spatial information score, which was non-significant even at the uncorrected significance level (p_1-tailed_= 0.162), remained non-significant (p_corrected_= 0.159). In sum, while all three response-based spatial information scores survived the multiple comparisons correction, this was not the case for the location-based scores.

To further test the robustness of response-based RSA effects in RSC, we conducted a searchlight analysis (seeSupplementary Information,Section 2.4for detailed methodology and results). This method is equivalent to performing RSA across the entire search volume covered by the functional scanning. Each voxel was assigned with a value corresponding to the RSA effect. Using a nonparametric permutation-based one-sample t test (Nichols & Holmes, 2002), with a group-level anatomical mask comprising all our ROIs (MTL + RSC) for small volume correction, we found that RSC was involved in all three response-based RSA effects (within landmark, within motion, and between cue; cluster-level inference, ps_FWE,1-tailed_< 0.007). These results further corroborate the robustness of our finding of cue-independent response-based spatial representations in this area.

Collectively, these findings indicate that response-based, cue-independent spatial representations in RSC are robust.

#### 3.2.5 Validity of collapsing across environments and days in calculating RSA effects

In the main analysis, we averaged out the environment factor, as it was not our primary focus. To assess the validity of this approach, we tested whether the response-based RSA effects were generalizable across the nature and city environments. The setup was identical to the main analysis, but now we considered the three types of environment relationships separately (within nature, within city, and between environment) for each type of cue relation (within landmark, within motion, and between cue), resulting in nine spatial information scores in total. The results showed that all three between-environment scores were significantly greater than 0 (t’s > 2.6, p’s_1-tailed_< 0.01, BF’s_10_> 7; pink bars inFig. 9a), indicating that the RSA effects were generalizable across environments. Moreover, for each cue relation, there was no significant difference between the nature and city scores. In sum, these findings support our approach of averaging out the environment factor in the main analysis.

**Fig. 9.**
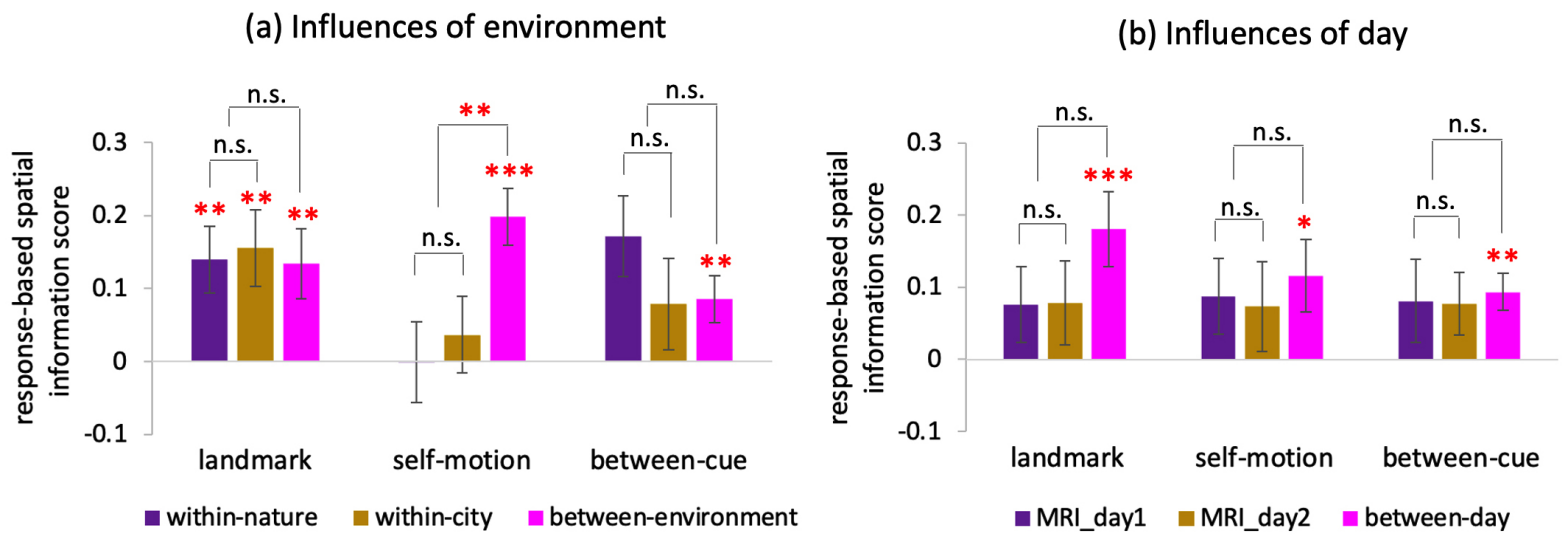
Influences of environment and day on RSA effects in RSC. (a) Influences of environment on response-based RSA effects in RSC. Plotted are response-based spatial information scores for each environment relation (within nature, within city, and between cue) and cue relation (within landmark, within motion, and between cue). (b) Influences of day on response-based RSA effects in RSC. Plotted are response-based spatial information scores for each day relation (MRI_day1, MRI_day2, and between day) and cue relation (within landmark, within motion, and between cue) “*”—p < 0.05, “**”—p < 0.01, “***”—p < 0.001. When testing whether the spatial information score was greater than 0, the one-tailed t test was adopted, because we had a specific directional hypothesis that pattern similarity should increase as inter-response distance decreased. When testing the difference between two spatial information scores, the two-tailed t test was adopted due to the lack of a specific directional hypothesis. Error bars represent±SE.

In the main analysis, in addition to averaging out the environment factor, we also combined data from the 2 scanning days together. We assessed the validity of this approach using the same method. The results showed that all three between-day scores were significantly greater than 0 (t’s > 2.2, p’s_1-tailed_< 0.02, BF’s_10_> 3.5; pink bars inFig. 9b), indicating that the RSA effects were generalizable across scanning days. Moreover, for each cue relation, there was no significant difference between the MRI_day1 and MRI_day2 scores. In sum, these findings support our approach of combining data across the 2 scanning days in the main analysis.^†^

#### 3.2.6 Spatial relationship between fMRIa and RSA effects within RSC

The preceding results demonstrate that RSC contained RSA-based spatial representations for both landmarks and self-motion cues, which were driven by response and generalizable between the cue types. Our previous study, which analyzed the same dataset, demonstrated that RSC contained fMRIa-based cue-specific spatial representations; specifically, the voxel-to-voxel pattern of adaptation was distinct between the cue types (Fig. 4b). Together, these findings raise the question of whether fMRIa-based and RSA-based spatial representations were spatially dissociable. If fMRIa and RSA effects overlapped anatomically, we would expect voxels with higher fMRIa effect to display stronger RSA effects. Since our results had shown that in RSC, fMRIa and RSA effects were driven by location (Chen et al., 2024) and response, respectively, we conducted this analysis using location-based fMRIa effects and response-based RSA effects.

**Fig. 10.**
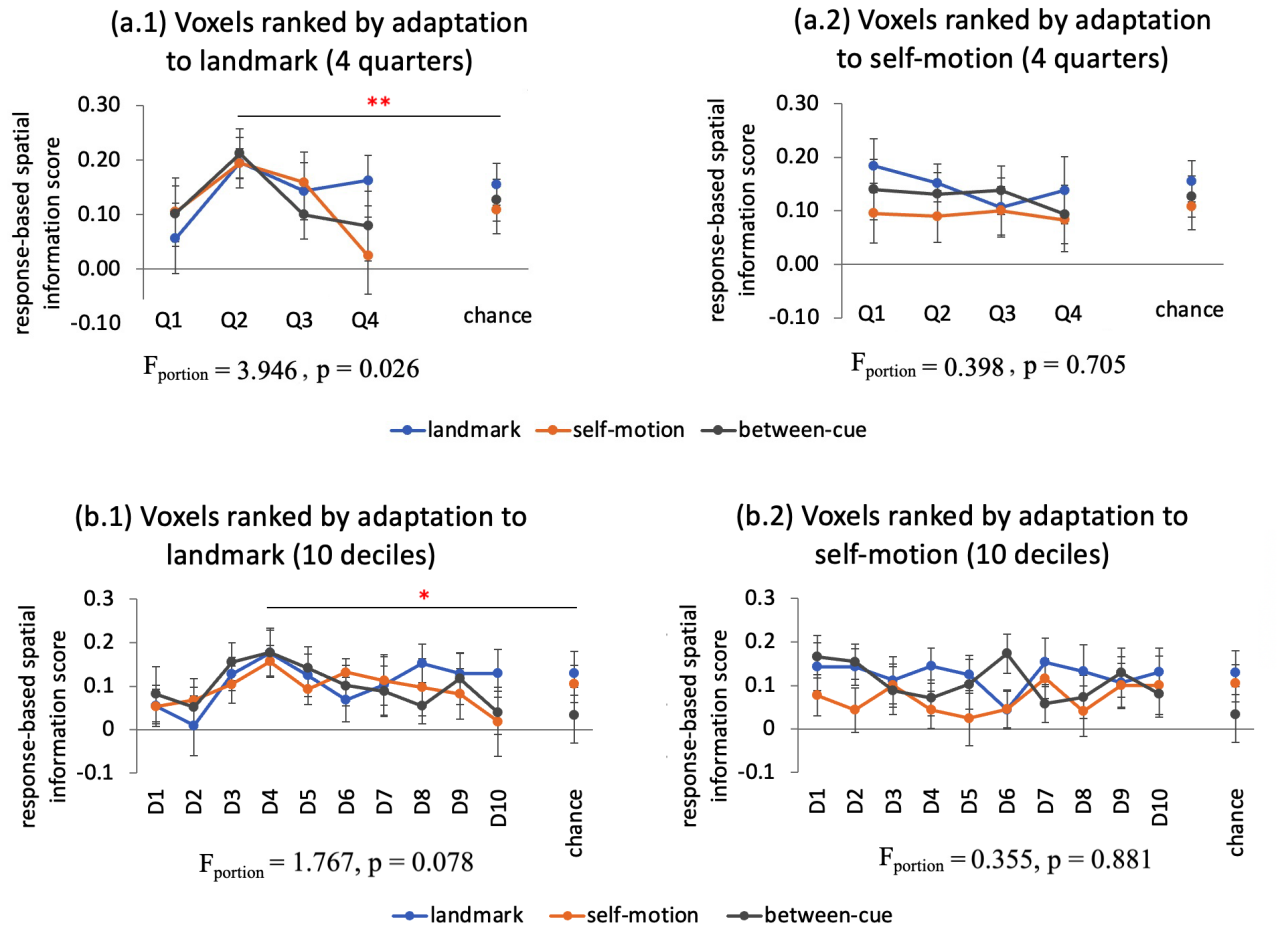
Spatial relationship between RSA and fMRIa effects in RSC. (a) Retrosplenial voxels were ranked by the magnitude of the mean fMRIa effect for landmarks (a.1) or self-motion cues (a.2), from low to high. The ranked voxels were then divided into four quarters. For example, voxels in the 1^st^quarter (Q1) showed lowest levels of adaptation. Response-based spatial information scores were calculated for each quarter separately. The empirical chance levels for the three scores (within landmark, within motion, and between cue) were also displayed. (b) The procedure was the same as (a), but the voxels were divided into 10 equally sized deciles instead of 4 quarters. “*”—p < 0.05, “**”—p < 0.01, “***”—p < 0.001. Error bars represent±SE.

First, we ranked the retrosplenial voxels by the magnitude of mean fMRIa effect in the landmark condition, from low to high (Fig. 10a.1, left panel). The ranked voxels were divided into four quarters, and the response-based spatial information scores were calculated for each quarter separately. The total number of voxels in each quarter ranged from 163 to 354 across participants, with a mean of 258, ensuring sufficient data points for calculating activation pattern similarity. We subjected these scores to a repeated-measures ANOVA test with quarter (Q1 vs. Q2 vs. Q3 vs. Q4) and score type (within landmark vs. within motion vs. between cue) as independent variables. The main effect of score type was not significant (F(2, 38) = 0.085, p = 0.919,$\eta_{\text{p}}^{2}$= 0.004, BF_10_= 0.113), nor was the interaction effect between quarter and score type (F(6, 114) = 1.093, p = 0.364,$\eta_{\text{p}}^{2}$= 0.054, BF_10_= 0.141). However, the main effect of quarter was significant (F(1, 19) = 3.946, p = 0.026,$\eta_{\text{p}}^{2}$= 0.172, BF_10_= 1.383). The second quarter exhibited the greatest spatial information scores, which were significantly greater than the other three quarters (vs. Q1, t(19) = 3.085, p = 0.006; vs. Q3, t(19) = 2.579, p = 0.018; vs. Q4, t(19) = 2.816, p = 0.011). Furthermore, the scores for the second quarter were also significantly greater than chance levels (F(1, 19) = 12.836, p = 0.002,$\eta_{\text{p}}^{2}$= 0.403, BF_10_= 6.962), whereas the scores for the other quarters were not significantly different from chance levels (F’s < 2.6, p’s > 0.12,$\eta {\text{s}}_{\text{p}}^{2}$< 0.12, BF’s_10_< 0.55). These results indicate that voxels with relatively lower landmark adaptation effects (i.e., voxels in the second quarter) exhibiting stronger RSA effects than other voxels.

Next, we ranked the retrosplenial voxels by the mean fMRIa effect for self-motion cues (Fig. 10a.2, right panel). The main effect of quarter was not significant (F(1, 19) = 0.398, p = 0.705,$\eta_{\text{p}}^{2}$= 0.021, BF_10_= 0.049), nor was the main effect of score type (F(2, 38) = 0.581, p = 0.564,$\eta_{\text{p}}^{2}$= 0.030, BF_10_= 0.195) or the interaction effect (F(6, 114) = 0.281, p = 0.913,$\eta_{\text{p}}^{2}$= 0.015, BF_10_= 0.024). For all four quarters, the response-based spatial information scores did not differ significantly from chance levels (F’s < 3, p’s > 0.099,$\eta {\text{s}}_{\text{p}}^{2}$< 0.14, BF’s_10_< 0.4). These results suggest that the self-motion adaptation levels of the voxels were unrelated to the RSA effects.

Our previous report showed that the voxel-to-voxel adaptation pattern was distinct between the 2 scanning days even within the same cue type (Chen et al., 2024). Consequently, we ranked the retrosplenial voxels by the adaptation magnitude for each cue type on each scanning day; still, no significant main effects of quarter were observed (F’s < 1.12, p’s > 0.35, ηs_p_^2^< 0.06, BF’s_10_< 0.2). Additionally, our previous report also showed that the voxel-to-voxel adaptation pattern was distinct between the two environments for self-motion cues within the same scanning day. Hence, we ranked retrosplenial voxels by the magnitude of adaptation to self-motion cues in each environment and each day; again, no significant main effects of quarter were observed (F’s < 1.11, p’s > 0.35,$\eta {\text{s}}_{\text{p}}^{2}$< 0.06, BF’s_10_< 0.15). Finally, the pattern of results remained unchanged when we divided the voxels into 10 equally sized deciles instead of four quarters (Fig. 10b). Note that the number of voxels in each decile ranged from 65 to 142 across participants, with a mean of 103, ensuring sufficient data points for calculating activation pattern similarity.

In summary, we found that retrosplenial voxels higher in adaption did not necessarily exhibit stronger RSA effects than other voxels. In most of the cases, there were no significant influences of adaptation magnitude on RSA effects. There was even one instance where the opposite happened, that is, voxels relatively lower in landmark adaptation displaying stronger RSA effects (Fig. 10a.1). Taken together, these findings suggest that RSA-based and fMRIa-based spatial representations were likely anatomically separable within RSC.

**Fig. 11.**
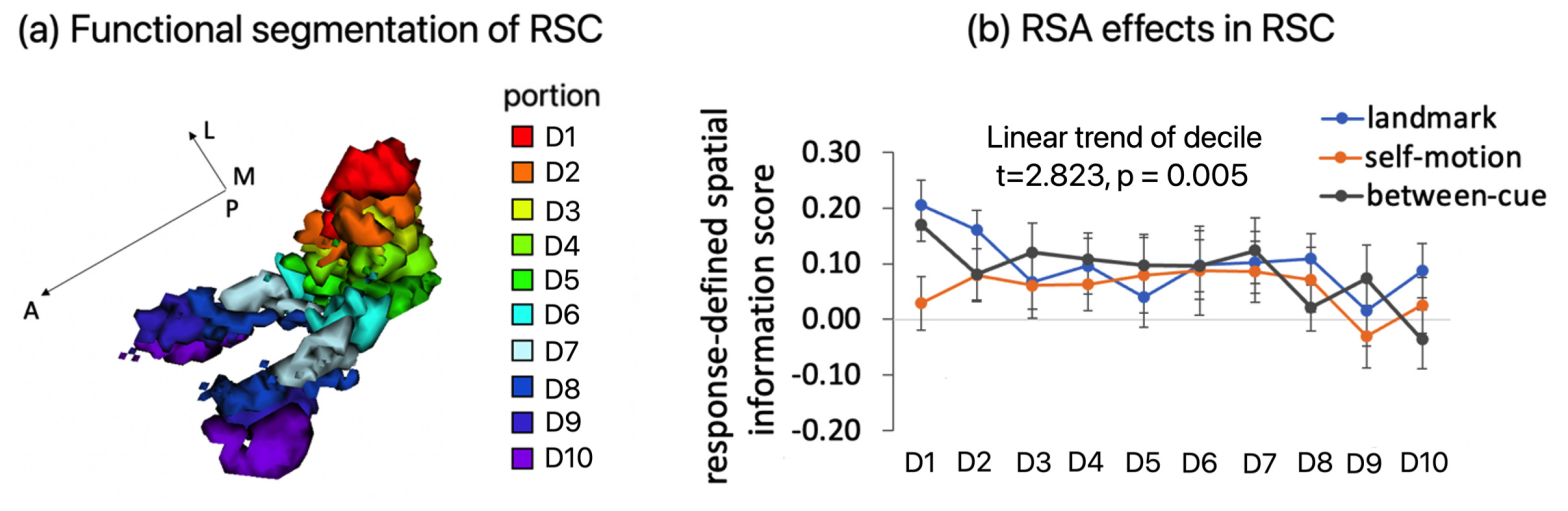
Analysis of spatial distribution of response-based RSA effects in RSC. (a) Displayed is the RSC of an example participant’s brain. RSC was divided into 10 equally sized deciles, based on its dominant connectopy (seeChen et al., 2024for detailed methodology). (b) Response-based spatial information scores were calculated separately for each of the 10 retrosplenial deciles. The scores are plotted as a function of retrosplenial decile. Error bars represent±SE.

However, these findings do not determine the spatial scale at which RSA-based and fMRIa-based spatial representations were anatomically separate. Our previous work showed that RSC exhibited a dominant connectopy along its long axis (Haak et al., 2018;Fig. 11a) and that fMRIa-based spatial representations in RSC were stronger in the eight posterior deciles than the two anterior deciles (figure 7 inChen et al., 2024). If RSA- and fMRIa-based spatial representations were segregated at a coarse spatial scale, we would expect distinct distribution patterns across retrosplenial subregions for these two types of spatial representations. Conversely, if fMRIa- and RSA-based spatial representations shared a similar distribution pattern across retrosplenial subregions, this would suggest that these two types of representations were likely intermixed at a relatively high spatial frequency.

To address this question, we calculated response-based spatial information score for each retrosplenial decile along the long axis of RSC (Mattar et al., 2018), which was then subjected to a repeated measures ANOVA, with decile and score type (within landmark vs. within motion vs. between cue) as independent variables. As shown inFigure 11b, the main effect of score type was not significant (F(2, 38) = 0.906, p = 0.413,$\eta_{\text{p}}^{2}$= 0.046, BF_10_= 0.106), neither was the interaction between decile and score type (F(18, 342) = 0.901, p = 0.578,$\eta_{\text{p}}^{2}$= 0.045, BF_10_= 0.014). Although the main effect of decile was not significant (F(9, 171) = 1.293, p = 0.244,$\eta_{\text{p}}^{2}$= 0.064, BF_10_= 0.028), the linear trend of decile was significant (t(171) = 2.823, p = 0.005). Planned contrast analysis revealed significantly stronger RSA effects in the eight posterior deciles than the two anterior deciles (t(171) = 2.874, p = 0.005), while the eight posterior deciles did not differ in RSA effects (F(7, 133) = 0.495, p = 0.837,$\eta_{\text{p}}^{2}$= 0.025, BF_10_= 0.109)—a pattern consistent with the fMRIa effects in our previous report (Chen et al., 2024).

In sum, our findings suggest that fMRIa- and RSA-based spatial representations are likely intermingled at a relatively high spatial frequency across RSC, providing further insight into their anatomical separability. Additionally, the relative uniform RSA-based spatial coding across the RSC (i.e., no differences among the eight posterior deciles of RSC) suggests that RSA-based spatial coding is broadly distributed within this region. Finally, our previous work indicates that landmark-specific and self-motion-specific fMRIa-based spatial representations may also be intermingled at a fine spatial frequency and widely distributed across RSC (Chen et al., 2024). Taken together, findings from our two investigations imply that the three different types of spatial representations—landmark-specific fMRIa, self-motion-specific fMRIa, and cue-independent RSA—are all intermingled at relatively fine spatial scales and relatively dispersed throughout RSC.

#### 3.2.7 Specificity of RSA-based spatial coding in RSC

The preceding analyses demonstrated RSA-based spatial coding in RSC was present for both cue types, driven by response rather than stimulus, and generalizable between cue types in terms of response. Here, we conducted analyses to further characterize the nature of this coding. Specifically, we assessed specificity of this coding by examining whether it was influenced by a variety of factors different from location and response, including temporal distance between location occupation events, traveled time, the passive movement phase preceding the location occupation phase, and path length.

To preview, we found that the response-based spatial coding in RSC was not confounded by temporal distance between location occupation events or traveled time.

For the passive movement phase preceding the location occupation phase, although the RSA effects for individual cues were unaffected by the modeling of this phase, accounting for this phase was necessary to unveil the cue-generalizable component of the response-based spatial coding in RSC. This finding offers methodological guidance for future investigation on cue specificity/generalizability of spatial coding using fMRI.

Regarding path length, analyses of individual cues revealed that retrosplenial coding was driven by the perceived allocentric position rather than by the path leading to the test location, indicating allocentric positional coding. However, the evidence is ambiguous regarding whether the cue-generalizable component of the positional coding was driven by perceived allocentric position or the path length, indicating a mixture of egocentric and allocentric elements in the representations.

##### 3.2.7.1 Temporal distance versus recognized location

To investigate whether the response-based positional coding in RSC was confounded by the temporal distance between the current location occupation event and the previous one (i.e., the interstimulus interval), we controlled for this factor using the same method for disentangling true location and response location (seeFig. 7a). We found that the response-based RSA effects remained significant after temporal distance has been accounted for in RSC: lumped score, t(19) = 4.075, p_1-tailed_< 0.001, BF_10_= 107; within landmark, (t(19) = 4.375, p_1-tailed_< 0.001, BF10 = 195); within motion, t(19) = 3.044, p_1-tailed_= 0.003, BF_10_= 14.309; between cue, t(19) = 2.852, p_1-tailed_= 0.005, BF_10_= 10.014. These findings indicate that the response-based positional coding observed in RSC was not confounded by temporal distance. This finding was expected because the correlation between inter-response spatial distance and temporal distance was minimal (Pearson r, mean = 0.033, SD = 0.018).

##### 3.2.7.2 Traveled time versus recognized location

To investigate whether the response-based positional coding in RSC was confounded by traveled time, we controlled for this factor using the same method for disentangling true location and response location (seeFig. 7a). We found that the response-based RSA effects remained significant after traveled time has been accounted for in RSC: lumped score, t(19) = 4.682, p_1-tailed_< 0.001, BF_10_= 361; within landmark, t(19) = 3.835, p_1-tailed_< 0.001, BF_10_= 66.319; within motion, t(19) = 2.941, p_1-tailed_= 0.004, BF_10_= 11.786; between cue, t(19) = 2.573, p_1-tailed_= 0.009, BF_10_= 6.056. These findings indicate that the response-based positional coding observed in RSC was not confounded by traveled time. This finding was expected because inter-response spatial distance and traveled time were only modestly correlated (Pearson r, mean = 0.226, SD = 0.038).

##### 3.2.7.3 Passive movement phase

So far, to account for the passive movement phase preceding the location occupation phase, we used separate regressors to model the passive movement phase in the first-level GLMs. However, the other two navigation phases “start” and “location arrival” were left unmodeled. Therefore, we constructed a new GLM (*GLM-RSA-response-revised*) in which the movement regressors were extended to encompass all three navigation phases (“start” + “movement” + “location arrival”) (see a similar control analysis in our previous report on location-based fMRIa effects;Chen et al., 2024). With this revised GLM, the pattern of results remained unchanged. All three response-based spatial information scores remained significant: within landmark, t(19) = 3.152, p_1-tailed_= 0.003, BF_10_= 17.549; within motion, t(19) = 2.186, p_1-tailed_= 0.021, BF_10_= 3.131; between cue, t(19) = 3.522, p_1-tailed_= 0.001, BF_10_= 35.827. These results indicate that our RSA findings associated with the location occupation phase were not influenced by how the preceding navigation experience was modeled in the first-level GLM.

To thoroughly evaluate the influences of the movement phase on our RSA findings, we also constructed a first-level GLM without modeling the movement phase (*GLM-RSA-response-NoPasMov*). The response-based within-cue spatial information scores remained significant (within landmark, t(19) = 3.319, p_1-tailed_= 0.002, BF_10_= 24.161; within motion, t(19) = 2.422, p_1-tailed_= 0.013, BF_10_= 4.652). However, the response-based between-cue spatial information score became marginally significant (t(19) = 1.704, p_1-tailed_= 0.052, BF_10_= 1.485). These results are interpretable. Given the evidently different sensory inputs between different cue conditions during passive movement and the slow temporal dynamics of the hemodynamic response, any cue-specific brain activity during the movement phase would have persisted into the subsequent location occupation phase, thereby obscuring the cue-generalizable component of the positional coding. Our findings suggest that it is necessary to account for the preceding cue-specific navigation experience to uncover the cue-generalizable component of the RSA-based positional coding in RSC.

##### 3.2.7.4 Path length versus recognized location

We evaluated whether the response-based positional coding in RSC could be disentangled from the length of the path leading to the test location. Given the randomized starting positions of movement across trials in both cue conditions, the path length was dissociated from the perceived location to a certain extent on a trial-by-trial basis for both cue types. This allowed us to evaluate whether the positional coding was primarily egocentric (i.e., driven by path length) or allocentric (i.e., driven by perceived location).^‡^

We employed*GLM-single-trial*, which modeled individual trials with separate regressors so that trial-specific disparity between path length and response could be accounted for. We estimated the unique contributions of response and path length to RSA effects, using the same approach for disentangling response and location (seeFig. 7a). This approach is versatile, as it can be used to disentangle any two variables that differ from each other on a trial-by-trial basis.

For the within-landmark spatial information score, the unique contribution of response remained significant after accounting for path length (t(19) = 4.307, p_1-tailed_< 0.001, BF_10_= 170.443); on the contrary, although the total contribution of path length was significant (t(19) = 2.577, p_1-tailed_= 0.009, BF_10_= 6.099), its unique contribution was not significant after accounting for response (t(19) = 1.333, p_1-tailed_= 0.099, BF_10_= 0.893). Similarly, for the within-motion spatial information score, the unique contribution of response was significant after accounting for path length (t(19) = 2.461, p_1-tailed_= 0.012, BF_10_= 4.975); on the contrary, although the total contribution of path length was significant (t(19) = 2.393, p_1-tailed_= 0.014, BF_10_= 4.427), its unique contribution was not significant after accounting for response (t(19) = 1.297, p_1-tailed_= 0.105, BF_10_= 0.852). However, in the case of between-cue spatial information score, the unique contribution of response was marginally significant after accounting for path length (t(19) = 1.637, p_1-tailed_= 0.059, BF_10_= 1.349); while the total contribution of path length was significant (t(19) = 2.430, p_1-tailed_= 0.013, BF_10_= 4.721), its unique contribution was marginally significant after accounting for response (t(19) = 1.684, p_1-tailed_= 0.054, BF_10_= 1.442). In brief, while the positional coding was predominantly driven by response rather than path length, the results do not conclusively determine the primary factor driving the cue-generalizable component of the coding.

To enhance statistical power, we also computed the lumped spatial information score based on all trials from all three cue conditions, treating them as originating from the same condition. The unique contribution of response was significant after accounting for path length (t(19) = 3.397, p_1-tailed_= 0.002, BF_10_= 28.058 > 10). Path length’s total contribution was significant (t(19) = 3.352, p_1-tailed_= 0.002, BF_10_= 25.741), and its unique contribution remained significant after accounting for response (t(19) = 1.992, p_1-tailed_= 0.030, BF_10_= 2.275), albeit with a smaller effect size compared with response.

In summary, the RSA-based positional coding in RSC predominantly adhered to an allocentric reference frame, because it was primary driven by the allocentric position of the target location as retrieved by the participant. However, there existed a small degree of egocentric positional coding related to path length. Furthermore, we encountered ambiguity in disentangling response and path length in the between-cue spatial information score, suggesting inconclusive evidence regarding the spatial reference frame defining the cue-generalizable component of the positional coding in RSC. These findings align with prior research indicating that RSC encodes spatial information in both egocentric and the allocentric reference frames (Chrastil et al., 2015;Mao et al., 2017), suggesting its role in transforming spatial information between these reference frames (Byrne et al., 2007).

#### 3.2.8 Results of hippocampus

Our previous report has shown that the hippocampus was strongly connected with RSC and also exhibited a tendency of cue-specific location-based adaptation (Chen et al., 2024). In addition, prior studies in non-human animals have intensively investigated cue specificity/generalizability of positional coding in the hippocampus (Geva-Sagiv et al., 2016;Markus et al., 1995;Quirk et al., 1990;Radvansky et al., 2021; to name a few). Additionally, prior studies have demonstrated strong functional coupling between RSC and hippocampus (Mao et al., 2018;Wyss & Van Groen, 1992). There, we conducted analyses to test RSA effects in the hippocampus, which, though being exploratory, could provide valuable insights for inter-species comparisons.

As illustrated inFigure 12andTables S2–S3, the hippocampus exhibited a pattern of results similar to those observed in RSC. First, we observed significant response-based spatial information scores for both landmarks (t(19) = 2.254, p_1-tailed_= 0.018, BF_10_= 3.50) and self-motion cues (t(19) = 4.815, p_1-tailed_< 0.001, BF_10_= 471), which generalized across cue types in terms of response (t(19) = 3.147, p_1-tailed_= 0.003, BF_10_= 17.362) (Fig. 11a). Unlike RSC, however, stimulus and behavior could not be unambiguously disentangled in the RSA effects (Fig. 11b; lumped spatial information score, unique contribution of response, t(19) = 1.201, p_1-tailed_= 0.122, BF_10_= 0.755; unique contribution of location, t(19) = 0.244, p_1-tailed_= 0.405, BF_10_= 0.282). Second, the response-based RSA effects were relatively concentrated in the subiculum (Fig. 11d). Third, while the reconstructed neural space exhibited a structure parallel to the behavioral performance pattern (Fig. 11d, left), it also significantly resembled the physical space in the landmark condition (Fig. 11d, right), indicating again that stimulus and behavior were less dissociated compared with RSC. Surprisingly, unlike RSC, the RSA-based positional coding in the hippocampus predominantly adhered to the egocentric reference frame: the unique contribution of response was not significant after accounting for path length (lumped score, t(19) = 1.408, p_1-tailed_= 0.088, BF_10_= 0.984); in contrast, the unique contribution of path length was significant after accounting for response (lumped score, t(19) = 3.117, p_1-tailed_= 0.003), with the Bayes factor indicating strong evidence for the alternative hypothesis (BF_10_= 16.423). Like RSC, the unique contribution of response was significant after accounting for temporal distance between location occupation events (lumped score, t(19) = 2.967, p_1-tailed_= 0.004, BF_10_= 12.378) and traveled time (lumped score, t(19) = 3.300, p_1-tailed_= 0.002, BF_10_= 23.280), indicating the response-based positional coding in the hippocampus was not confounded by these two factors. Finally, like RSC, there were not significant differences between the left and right hippocampus in response-based spatial coding (F(1, 19) = 1.577, p = 0.224,$\eta_{\text{p}}^{2}$= 0.077).

**Fig. 12.**
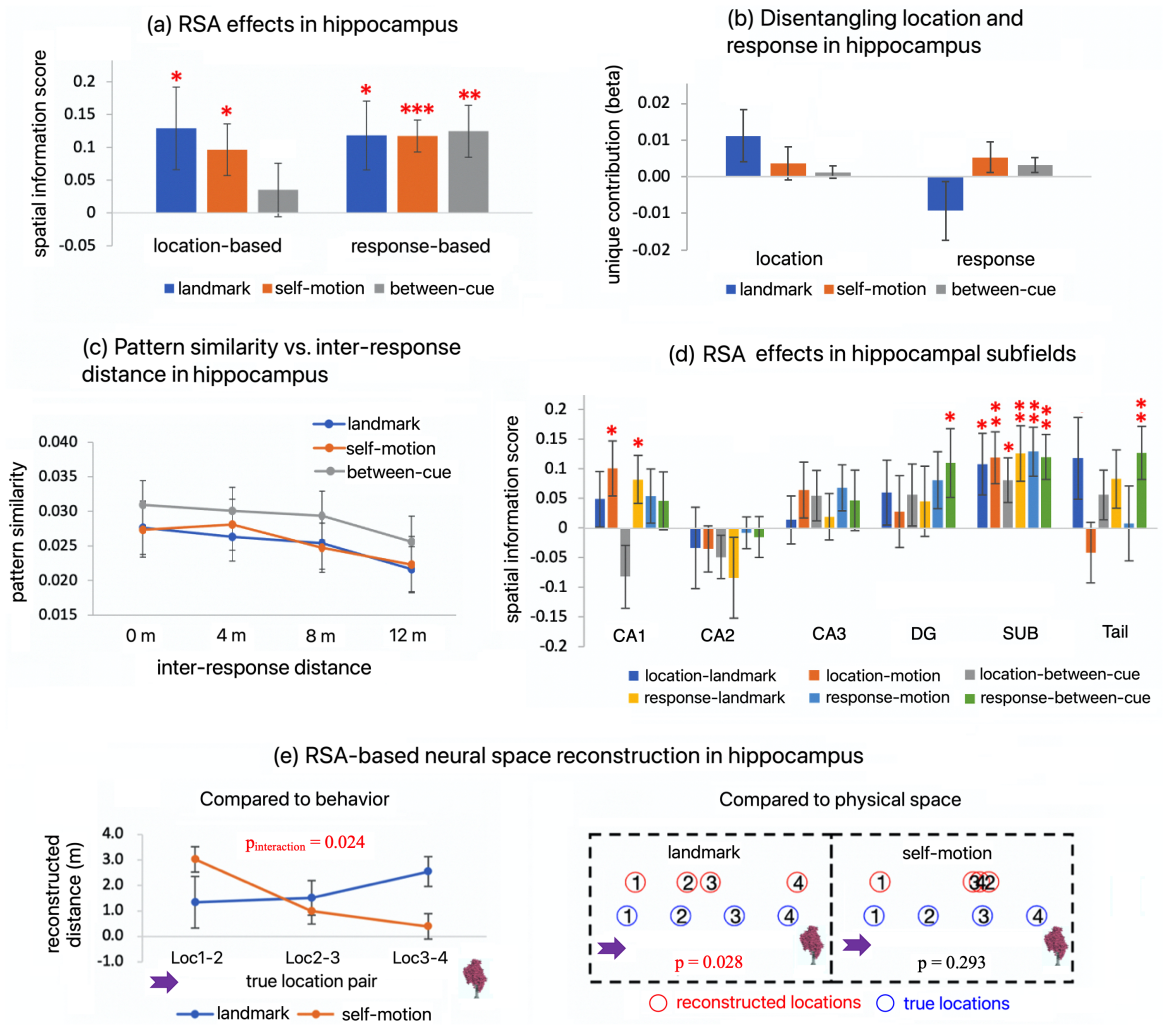
Results of the hippocampus. (a) Spatial information scores based on location and response for landmark, self-motion, and between cue. (b) Unique contributions of location and response to RSA effects for landmark, self-motion, and between cue. (c) Visualization of the response-based RSA effects in (a) Multi-voxel activation pattern similarity is plotted as a function of inter-response distance for landmark, self-motion, and between cue. (d) Location-based and response-based RSA effects in hippocampal subfields. It is worth mentioning that in the subiculum, all the six scores survived multiple comparisons correction across the six one-sample t tests (p’s_corrected_< 0.05). (e) RSA-based neural space reconstruction analysis. In the left panel, the neural space was compared with behavioral performance pattern (Fig. 4). The interaction between cue type and the linear trend of adjacent location pair was significant (p_interaction_= 0.024). In the right panel, the group-level neural space significantly resembled the physical layout of the four test locations in the landmark condition (p = 0.028), but not in the self-motion condition (p = 0.293). Numbers in the circles denote the four test locations (e.g., “1” indicates Loc1). “*”—p_1-tailed_< 0.05, “**”—p_1-tailed_< 0.01, “***”—p_1-tailed_< 0.001. When testing whether the spatial information score was greater than 0, the one-tailed t test was adopted, because we had a specific directional hypothesis that pattern similarity should increase as inter-response distance decreased. Error bars represent±SE.

For completeness, we also presented RSA results for other ROIs, detailed in the Supplementary Information (Tables S2 and S3). Besides RSC and hippocampus, exploratory analyses revealed evidence of cue-independent positional coding in terms of response in the parahippocampal cortex (PHC). Akin to the hippocampus, positional coding in PHC was egocentric rather than allocentric: the unique contribution of response was not significant after accounting for path length (lumped spatial information score, t(19) = 1.129, p_1-tailed_= 0.136, BF_10_= 0.692), while the unique contribution of path length was significant after accounting for response (lumped score, t(19) = 1.956, p_1-tailed_= 0.033, BF_10_= 2.170). However, response and stimulus could not be clearly dissociated in the positional coding in the PHC: the unique contribution of response after accounting for location was not significant (lumped score, t(19) = 0.989, p_1-tailed_= 0.168, BF_10_= 0.587), neither was the unique contribution of location after accounting for response (t(19) = 0.465, p_1-tailed_= 0.324, BF_10_= 0.342).

## 4 Discussion

In the current study, we analyzed ultra-high-field fMRI data from human navigating in a desktop virtual reality environment using either landmarks alone or visual self-motion cues alone. While our previous fMRI adaptation (fMRIa) analysis demonstrated that RSC contained spatial representations for both cue types (Chen et al., 2024), here we adopted a complementary approach—representational similarity analysis (RSA). We observed RSA-based spatial representations for both cue types in RSC, with representational similarity between locations defined by the same cue type scaling with perceived spatial proximity. This RSA-based coding differed from the fMRIa-based coding in two main aspects. First, while the fMRIa-based coding showed stronger association with physical inputs (i.e., participant’s actual location), RSA-based coding showed stronger association with behavior (i.e., participant’s self-reported location). Second, while the fMRIa-based coding was cue specific (i.e., voxel-to-voxel adaptation patterns differed between cues), the RSA-based coding was cue independent, with representational similarity between locations defined by different cue types also scaling with perceived spatial proximity. Taken together, to our knowledge, our study is the first to demonstrate the coexistence of cue-specific and cue-independent spatial representations in the human RSC.

When changing sensory information in a given environment, previous studies have reported cue-independent spatial representations in RSC in both humans (Huffman & Ekstrom, 2019) and rodents (Mao et al., 2017).Huffman and Ekstrom (2019)manipulated self-motion cues by varying the amount of body-based information across different virtual environments, where participants learned spatial locations. Subsequent retrieval of these locations during fMRI scanning recruited similar brain networks, including RSC, across environments. Their findings provide evidence for cue-independent spatial representations. Similarly,Mao et al. (2017)manipulated spatial cues by switching lights on and off. They found that the collective firing pattern of place-sensitive cells in RSC remained stable across different lighting conditions, suggesting cue-independent spatial representations in this region. However, in both studies, different cue types were not fully dissociated across conditions. InMao et al. (2017), body-based self-motion cues were available in both lighting conditions, whileHuffman and Ekstrom (2019)maintained visual information across all self-motion conditions. Consequently, the reported cue-independent spatial representations may have been driven by shared spatial information, rather than representing a genuine cue-independent spatial code that transcends the sensory sources of spatial information.

To overcome these limitations, our experimental design ensured a complete dissociation between landmarks and self-motion cues. This manipulation proved effective, as evidenced by differential patterns of behavioral accuracy along the linear track in different cue conditions. Specifically, in the landmark condition, performance improved as the test location neared the landmark, while the opposite pattern was observed in the self-motion condition. This finding can be explained by the decreasing spatial accuracy of landmark-based navigation as distance from the landmark increases (Chamizo et al., 2006;Chen et al., 2019). Meanwhile, path integration becomes more prone to errors as the navigator moves away from the reference point (Stangl et al., 2020). These behavioral findings indicating that our experimental manipulation effectively elicited distinct navigational strategies reliant on landmarks versus self-motion cues. Hence, our finding of RSA-based cue-independent spatial representations in RSC is unlikely to be confounded by overlapping spatial information across different cue conditions. Instead, this finding likely signifies an abstract positional coding that surpasses diverse sensory sources of spatial information.

Crucially, given that we also detected fMRIa-based cue-specific representations in RSC using the same dataset (Chen et al., 2024), it is possible that the two prior studies might have overlooked parallel cue-specific spatial representations that could manifest in a different form of neural activity (Huffman & Ekstrom, 2019;Mao et al., 2017). Our study underscores the importance of leveraging complementary neural phenomena to achieve a more comprehensive understanding of the neural operations supporting the formation of coherent cognitive maps.

Why would cue-specific and cue-independent spatial representations coexist in RSC? From a behavioral perspective, our task comprised both a sensory component and a long-term memory component, as participants had to compare sensory inputs with memory traces of spatial locations to determine where they were located. The sensory component involved deriving positional estimates from the perception of cue-specific spatial inputs. In contrast, the long-term memory component involved forming and retrieving memory traces of the four test locations. This component was cue independent because the test locations remained the same regardless of the cue type used. Although the behavioral performance profile along the linear track differed between the landmark and self-motion conditions (Fig. 4a), additional analysis revealed a strong across-participant correlation in behavioral accuracy between the two cue conditions (r = 0.696, N = 20, p < 0.001). This result means that those who performed better with landmarks also performed better with self-motion cues. This finding indicates that the two cue conditions might share certain cognitive components, which likely correspond to the common long-term memory traces of the test locations.

Consistent with the behavioral findings, our fMRI results revealed contrasting properties of fMRIa and RSA effects in RSC: while fMRIa effects were cue specific and associated with stimulus input (i.e., objective location), RSA effects were cue independent and linked to behavior (i.e., retrieved spatial memory). The coexistence of stimulus-oriented fMRIa effects and behavior-oriented RSA effects in RSC suggests that this region is crucially involved in the neural process of comparing perceptual inputs with stored memory traces to make navigation decisions (Alexander et al., 2023).

Notably, our fMRI results align broadly with previous studies reporting distinct properties of fMRIa and MVPA effects (RSA effects included). These studies focused on brain regions associated with scene and object processing, which are also involved in spatial navigation (Chen et al., 2019;Diersch et al., 2021;Epstein, 2008;Julian et al., 2016). fMRIa and MVPA effects differ in two main aspects: stimulus selectivity and relationship with behavior. First, regarding stimulus selectivity, fMRIa effects are sensitive to viewpoint changes of scenes in brain regions such as the parahippocampal place area (PPA), retrosplenial complex, and lateral object complex (LOC) (Epstein et al., 2003). In contrast, MVPA effects code spatial locations of the scenes independent of facing direction in regions such as the presubiculum, retrosplenial complex, and parietal-occipital sulcus (Vass & Epstein, 2013); additionally, the presubiculum contains MVPA-based representations for facing direction independent of spatial locations of the scenes (Vass & Epstein, 2013). Consistent with both streams of evidence,O’Connell et al. (2018)compared color photographs and line drawings of natural scenes, and demonstrated concurrent stimulus-specific fMRIa-based coding and stimulus-independent MVPA-based coding of scenes in the same brain regions, including the retrosplenial complex, PPA, and OPA. These results demonstrate that while fMRIa effect is sensitive to lower level physical features of stimuli, MVPA effects reflect abstract coding of the target stimulus dimension by remaining insensitive to changes in other stimulus dimensions. The stimulus selectivity of fMRIa effects in human studies is reminiscent of the stimulus selectivity observed in neuronal adaptation in electrophysiological studies (De Baene & Vogels, 2010;Liu et al., 2009;Sawamura et al., 2006). Second, regarding relationship with behavior, the scene-related fMRIa effect in PPA is unaffected by top-down cognitive operations, such as task requirements that typically influence behavioral performance (Xu et al., 2007). In contrast, the MVPA effect in LOC reflects behavioral confusability among stimulus items (Hatfield et al., 2016), and better behavioral performance in discriminating stimulus items corresponds to more distinct MVPA-based neural representations in regions such as the retrosplenial complex (Koch et al., 2020;Walther et al., 2009), alEC (Bellmund et al., 2019), PPA (Walther et al., 2009), and LOC (Walther et al., 2009;Williams et al., 2007). The close relationship between MVPA effects and behavior in human studies is reminiscent of findings that the collective activity of neuronal populations in the hippocampus (Saleem et al., 2018) and RSC (Alexander & Nitz, 2015) reflects overt behavior in electrophysiological studies. However, this link should be interpreted with caution due to the unclear relationship between BOLD signals and neuronal activity (Arthurs & Boniface, 2002).

Having established that RSC contained concurrent cue-specific and cue-independent spatial representations associated with stimulus and behavior, respectively, one critical question arises: why did this intriguing phenomenon occur in RSC? The anatomical and functional characteristics of RSC make it well suited to support navigation behavior by mediating the interplay between sensory processing and long-term memory functions (Alexander et al., 2023). On one hand, RSC’s connections with brain regions that process sensory information of landmarks and self-motion cues are fundamental to this role (Vann et al., 2009). In primates, RSC is anatomically connected with occipital regions and V4, whose human homologue, hV4, is hypothetically situated in the lingual gyrus, a region critical for landmark recognition (Pallis, 1955). Notably, human RSC demonstrates structural and functional connectivity with BA17 (Li et al., 2018), whose activity distinguishes different object sizes (Schwarzkopf et al., 2011); this size-distinguishing signal can potentially aid in gauging distance to a landmark based on its perceived size (Howard, 2002). Moreover, human RSC displays functional connectivity with optic-flow-responsive areas such as V3A, V6, and hMT+ during path integration (Sherrill et al., 2015). RSC also has dense connections with the thalamus (Vann et al., 2009), which is involved in spatial processing of both landmarks (Yoder et al., 2011) and self-motion cues (Arleo et al., 2013). On the other hand, RSC’s anatomical connectivity with MTL in primates (Vann et al., 2009) underscores its pivotal role in long-term memory functions. Human RSC also mediates the functional connectivity between MTL and the cortical areas of the default mode network, with this mediation correlating with episodic memory performance (Kaboodvand et al., 2018). Accordingly, RSC often serves as a repository for long-term memory traces relevant to spatial navigation (Diersch et al., 2021;Patai et al., 2019;Wolbers & Büchel, 2005). In brief, the interaction between the sensory and memory components makes RSC pivotal for the formation of coherent cognitive maps from various spatial inputs during navigation (O’Keefe & Nadel, 1978;Tolman, 1948).

How can the coexistence of cue-specific and cue-independent spatial representations in RSC refine our understanding of its functional architecture? First, we previously found that the voxel-to-voxel patterns of fMRIa effect based on objective location were spatially dissociated between landmarks and self-motion cues (Chen et al., 2024), suggesting the existence of distinct location-sensitive neuronal subpopulations processing sensory inputs from the two cue types, respectively. These neuronal subpopulations should display adaptation to stimulation from external spatial inputs. Second, we currently found that RSA effects based on subjective response were spatially generalizable between cue types, which is indicative of a location-sensitive neuronal subpopulation whose ensemble activity represented the navigator’s subjective recognition of spatial locations in a cue-independent manner. Crucially, this particular neuronal subpopulation should not display adaptation; otherwise, the voxel-to-voxel patterns of fMRIa effect would show spatial overlap between the cue types, thus eliminating the cue specificity observed in the voxel-to-voxel pattern of fMRIa effect. Third, RSA and fMRIa effects were independent at the voxel level, indicating that the non-adapting subpopulations reflecting subjective responses are anatomically separable from the adapting subpopulations encoding objective locations (Fig. 10). Finally, our findings also suggest that all these neuronal subpopulations were rather distributed along the long axis of RSC and intermingled with one another at high spatial frequencies. Collectively, our findings align with recent rodent studies suggesting that RSC contains distinct neuronal subpopulations, which respond differently to the adaptation manipulation (Brennan et al., 2020) and task engagement (Fischer et al., 2020). Our findings also suggest that adaptation can be a vital tool for distinguishing diverse neural units serving different computational purposes during navigation. Nevertheless, given the elusive relationship between BOLD signals and neuronal activity (Arthurs & Boniface, 2002), our interpretation is speculative at this point and awaits further rigorous investigations.

Although our findings suggest that RSC could be a candidate locus of spatial cue unification due to simultaneous cue-specific and cue-independent spatial representations in this region, the precise role RSC plays in this process remains to be determined. One hypothesis is that RSC performs the computations of cue unification (unification-in-RSC hypothesis). Alternatively, RSC might merely relay cue-specific spatial information to other brain regions for unification and subsequently receives cue-independent representations via feedback projections (unification-outside-RSC hypothesis). Arbitrating between these two hypotheses likely hinges on the connectivity structure within RSC (Alexander et al., 2023). We previously speculated that there might exist three separate neuronal subpopulations: two adapting subpopulations responding to the sensory inputs stemming from landmarks and self-motion cues, respectively, and a third non-adapting sub-population representing abstract spatial locations as retrieved by the navigator. The unification-in-RSC hypothesis would require strong interactions among these neuronal subpopulations within RSC. Conversely, limited interaction would support the unification-outside-RSC hypothesis. The unification-outside-RSC hypothesis also appears plausible, as RSA-based cue-independent spatial representations were observed not only in RSC but also in regions such as the hippocampus and PHC. Notably, the hippocampus may simultaneously contain fMRIa-based cue-specific spatial representations (Chen et al., 2024, see the Discussion section). Furthermore, the searchlight analysis revealed spatial coding in various brain regions, including the middle temporal gyrus and middle occipital gyrus (Table S5;Fig. S3;Chen et al., 2024, fig. E4). Further investigation is warranted to clarify the roles of RSC and other regions, such as hippocampus and PHC, in forming coherent cognitive maps and integrating various spatial cues during navigation, and it would be enlightening to interpret RSC’s spatial functions within the broader neural network of spatial navigation, emphasizing its interactions with other relevant regions rather than viewing it as an isolated processing module (Ekstrom et al., 2017).

Our findings not only illuminate the spatial cue unification process in the brain, but also provide insights into the mechanisms underlying fMRIa- and MVPA-based neural coding, a key topic in the fMRI literature. Currently, there are two main hypotheses: the input-versus-output hypothesis and the tuning-versus-clustering hypothesis.^§^

The input-versus-output hypothesis proposes that fMRIa effects reflect neural processing at the synaptic level, whereas MVPA effects capture contents of neuronal outputs (Epstein & Morgan, 2012). Our findings are partially consistent with this hypothesis. First, this hypothesis predicts that fMRIa and MVPA effects would be associated with stimulus and behavior, respectively, which is exactly what we observed in our studies. It is conceivable that the synaptic processing reflects the sensory information received by RSC from lower level cortical sensory areas, whereas neuronal outputs are more directly related to the behavior after RSC has incorporated sensory information with stored memory traces. Second, this hypothesis implies that fMRIa and RSA effects are dissociated within the same neuron, which predicts spatial overlap between these two types of effects. However, this prediction is contradicted by our finding that fMRIa and RSA effects appeared anatomically separable: voxels higher in fMRIa effect did not contribute more to RSA effects (Fig. 10).

We propose that separate neural subpopulations are responsible for stimulus-oriented coding and behavior-oriented spatial coding, which helps explain the coexistence of both fMRIa and RSA effects within RSC. This is particularly relevant, because RSC is hypothesized to mediate the bidirectional information flow between the parietal cortex and medial temporal lobe, as formalized in a well-known neural network model (Byrne et al., 2007). Relating this model to our task, it is conceivable that sensory information flows from the parietal cortex to MTL via RSC in a feedforward pathway, while behavior-related information is sent back to the parietal cortex from MTL in a feedback pathway. If a single group of neurons in RSC were to receive both stimulus-oriented information from the parietal cortex and behavior-oriented information from MTL, this neuron group would process both types of information at the synaptic level, making stimulus- and behavior-oriented coding inseparable. On the contrary, having distinct neuronal subpopulations for stimulus and behavior makes the coexistence of these neural representations plausible. Moreover, one potential benefit of this neuronal separation is that it readily allows for cue segregation within RSC, alongside cue unification. Cue segregation typically occurs when the sensory inputs from different cue types are considered to originate from discrepant spatial origins (French & DeAngelis, 2020;Körding et al., 2007). In this case, the cues should be separated rather than integrated. Future research will need to examine how the hypothesized sensory- and memory-driven neural subpopulations interact to support complex computations such as cue unification, segregation, and integration.

Contrary to our findings,Mattar et al. (2018)report that in the lateral occipital complex (LOC), voxels with higher fMRIa effects also exhibited greater RSA effects compared with those with lower fMRIa effects, suggesting that the same set of neural units was involved in both types of effects. This finding is consistent with the input-versus-output hypothesis and implicates neuronal adaptation as a mechanism for sharpening neural representations within the same neuronal population. The discrepancies betweenMattar et al. (2018)and our study indicate that the spatial relationship between fMRIa and MVPA effects may differ across brain regions.

The tuning-versus-clustering hypothesis is proposed byDrucker and Aguirre (2009). The authors found that the ventral LOC contained fMRIa-based representations of objects, while the lateral LOC contained RSA-based representations of objects. At the same time, they also found that the voxel tuning curve was rather broad over the object space in the ventral LOC, whereas it was much narrower in the lateral LOC. Based on these findings, they concluded that fMRIa effects reflect broad tuning curve of individual neurons, whereas RSA effects reflect clustering of neurons at a coarse spatial scale.

However, it may be premature to generalize properties of the voxel tuning curve to the neuronal tuning curve. In addition to the width of neuronal tuning curve, the evenness of spatial distribution of neurons across voxels also influences the voxel tuning curve. This is because the measurement unit in fMRI studies is voxel, and each voxel contains thousands of neurons. We run a simplified simulation to examine influences of the two factors on fMRIa and RSA effects. Detailed methodology and results are provided in Supplementary Information (Section 3). In brief, we found that the width of the neuronal tuning curve, which reflects representational overlap at the neuron level, influences fMRIa- and RSA-based effects in a similar manner, contradicting the tuning-versus-clustering hypothesis proposed byDrucker and Aguirre (2009). Particularly, for both types of effects, the emergence of spatial distance coding requires a moderately broad neuronal tuning, while the emergence of location identity coding requires a sharp neuronal tuning. In contrast, the evenness of the across-voxel distribution of neurons influences these two types of coding in opposite ways: a less even distribution of neurons leads to stronger MVPA effects but weaker fMRIa effects. However, the influence on fMRIa effects is minimal unless the distribution is highly uneven. These results are consistent with the tuning-versus-clustering hypothesis, in that MVPA effects benefit from relatively coarse spatial distributions of neurons across a brain region.

At a first glance, our finding that fMRIa effects were stimulus driven but not behavior driven seems to conflict with the turning-versus-clustering hypothesis’s proposition that fMRIa effects reflect the tuning of individual neurons. This proposition associates fMRIa effects to behavior, as sharper tuning curves correspond to less neuronal representation overlap among different stimulus items and thus better behavioral performance in distinguishing them. However, this contradiction is not necessarily true. As discussed in our previous paper, it is plausible that different parameters of the neuronal tuning curve influence different aspects of the fMRIa effect (Chen et al., 2024). For example, the means of the tuning curves are determined by objective location, which fit fMRIa-based spatial coding better than perceived location. Meanwhile, the standard deviations of the tuning curves represent the precision of sensory information, shaping the format and magnitude of the fMRIa effect, which in turn affects behavioral accuracy.

To test this hypothesis’s proposition that MVPA effects reflect coarse spatial clustering of neurons with our data, we re-computed the response-based spatial information scores using spatially smoothed beta images from the first-level GLM (with 3 mm isotropic FWHM) (Op de Beeck, 2010). In RSC, response-based spatial information scores remained significant: within landmark, t(19) = 4.661, p_1-tailed_< 0.001, BF_10_= 347; within motion, t(19) = 4.401, p_1-tailed_< 0.001, BF_10_= 206; between cue, t(19) = 3.679, p_1-tailed_< 0.001, BF_10_= 48.776. Given that spatial smoothing reduces differences among voxels, the persistence of RSA-based coding in RSC after spatial smoothing suggests that (i) the anatomical scale of location-sensitive neural units is rather coarser in RSC and (ii) uneven across-voxel distribution of neural units is a prerequisite for the emergence of RSA effects in a region, as posited by the tuning-versus-clustering hypothesis and demonstrated in our simulation analysis.

In summary, our findings are partially consistent with the existing two primary hypotheses regarding the neuronal mechanisms underlying fMRIa- and MVPA-based coding. Relating our findings to these hypotheses enhances our understanding of the neural processes driving these two types of neural coding. However, this understanding must be considered within the context of the specific brain region under investigation, as the underlying mechanisms may vary across different regions.

## 5 Conclusion

In the current study, we observed RSA-based cue-independent neural representations of spatial locations in RSC. Together with our previous finding of fMRIa-based cue-specific spatial representations in the same region, our study is the first to demonstrate the concurrent presence of cue-specific and cue-independent spatial representations in RSC. Our findings provide novel insights into a fundamental question in the spatial navigation literature: how the brain constructs coherent cognitive maps from various sensory sources of spatial information. Our findings suggest that RSC may play a crucial role in the unification of spatial cues, thereby facilitating the creation of coherent cognitive maps during navigation.

## Supplementary Material

Supplementary Material

## Data Availability

The data that support the findings of this study are available from the corresponding author, X.C., upon reasonable request. The data and code sharing adopted by the authors comply with the requirements of the funding body and the ethics approval of the local ethics committee.

## Footnotes

* To get a better idea of the reliability of our results, we calculated the amount of data excluded due to a perfect correlation between location and response. When the behavioral performance was perfect in both runs, the run pair was assigned a “NaN” value for the beta estimates (i.e., unique contributions of location and response) and excluded from further analysis. When all the participants were considered, for the within-landmark spatial information score, 64 out of 28 run pairs * 20 subjects = 560 run pairs (=11.43%) showed a 100% accuracy rate and were dropped from analysis. For the within-motion spatial information score, 18 out of 560 run pairs (=3.21%) were excluded. For the between-cue spatial information score, 31 out of 1280 run pairs (=2.42%) were excluded.

† Additionally, we also investigated potential hemispheric specificity of spatial coding in RSC by subjecting the response-based spatial information scores to a repeated-measures ANOVA test, with hemisphere (left vs. right) and score type (within landmark vs. within motion vs. between cue) as independent variables. The main effect of hemisphere was not significant (F(1, 19) = 0.293, p = 0.595,$\eta_{\text{p}}^{2}$= 0.015), nor was the interaction between hemisphere and score type (F(2, 38) = 1.154, p = 0.326,$\eta_{\text{p}}^{2}$= 0.031). The main effect of score type was not significant either (F(2, 38) = 0.610, p = 0.549,$\eta_{\text{p}}^{2}$= 0.057). These results indicate no hemispheric specificity of spatial coding in RSC.

‡ Egocentric spatial representations are defined within a reference frame centered on the moving navigator, reflecting first-person experience. In contrast, allocentric spatial representations are anchored in a reference frame centered on the external world and remain independent of the navigator’s position and head direction. Path integration is generally categorized as egocentric since it involves updating one’s position and head direction based on self movement. However, in our self-motion condition, the traveled path is distinctly egocentric, because its starting point is anchored to the navigator’s body. On the contrary, recognizing the test location required integrating self-motion inputs relative to an external reference point (i.e., the fixed red arrow, the anchoring point of path integration), thereby introducing an allocentric element. Moreover, prior work has demonstrated that path integration can operate within both allocentric and egocentric reference frames (He & McNamara, 2018). We thus conceptualize egocentric and allocentric spatial representations as a continuum rather than a strict dichotomy.

§ Epstein and Morgan (2012) also describe a third hypothesis, proposing that fMRIa effects reflect top-down influences of higher level cognitive functions, such as expectation (Summerfield et al., 2008). However, this hypothesis is challenged by a later study (Kaliukhovich & Vogels, 2011). Moreover, our study adopted randomized location sequences, excluding the impact of expectation. Therefore, we have, therefore, chosen not to discuss this hypothesis here.
