# Supplementary material for: Representational similarity analysis reveals cue-independent spatial representations for landmarks and self-motion cues in human retrosplenial cortex
Source: Imaging Neurosci (Camb). 2025 Mar 24;3:imag_a_00516. doi: 10.1162/imag_a_00516 (PMC12319769; doi:10.1162/imag_a_00516)
Supplement: Supplementary Material [file imag_a_00516-supp.pdf]

Supplemental Information

1. Supplemental Methods

1.1. Nonparametric permutation-based multiple comparisons correction

The measurements were submitted to a multiple comparisons correction approach that combines the nonparametric permutation-based maximum-t-statistic method and the Holm-Bonferroni method, to control the familywise type I error at 0.05. Specifically, first, in every permutation, every entry in each measurement was randomly multiplied by -1 or +1, and the t statistic was calculated for the permuted data of each measurement. Next, the maximum t statistic was obtained out of all the measurements. After 5000 permutations, we obtained a surrogate distribution of maximum t statistic, to which we compared the observed t statistic calculated from the actual data of each measurement. The corrected significance level (i.e., $p_{corrected}$) equaled to the proportion of values in the surrogate distribution of maximum t statistic that were greater than the observed t statistic.

This permutation procedure was performed iteratively, in that if the measurement with the lowest uncorrected p value survived the test, this measurement was deemed significant after multiple comparisons correction and was excluded from further analysis. Next, the remaining measurements were submitted to the same permutation test again. This procedure was repeated until the measurement with the lowest uncorrected p value did not pass the statistical significance threshold or no measurements were left for testing.

2. Supplemental Results

2.1. Re-examination of our first fMRI study (Chen et al., 2019)

We conducted the representational similarity analysis (RSA) to calculate the location-based spatial information scores for the ROIs in the medial temporal lobe (Table S1). In this experiment, participants first learned the fixed location of a target along a linear track. During the subsequent testing, they were passively transported to the four test locations in a predetermined de Bruijn sequence. At the test location, they needed to judge whether the target location was ahead or behind. Note that the response-based spatial information scores for the four test locations could not be calculated because participants were not asked to report the identity of the four test locations.

There were four cue conditions. In the “landmark high” condition, participants relied on the landmark cue only and the landmark was high in reliability. In the “landmark low” condition, participants relied on the landmark cue only and the landmark was low in reliability. In the “self-motion high” condition, participants relied on the optic flow cue only, which was high in reliability. In the “self-motion low” condition, participants relied on the optic flow cue only, which was low in reliability.

Regarding the entorhinal subregions, the results showed that the right pmEC exhibited location-based RSA effects in the landmark low condition, with the Bayes factor indicating moderate evidence for the alternative hypothesis (BF_{10} = 4.588). However, this effect is challenging to interpret, as the right pmEC did not exhibit spatial coding in the landmark high condition (BF_{10} = 0.135), suggesting that this area may not process landmark-related spatial information per se. It is possible that spatial coding observed in the right pmEC in the landmark low condition reflects participants’ reliance on path integration, given that landmark information was uninformative in this condition: because the four test locations were arranged along the linear track, on average the traveled distance differed to a certain degree among these locations.

Notably, the right pmEC did not exhibit significant location-based fMRIa effect in the same cue condition, meaning no concurrent fMRIa effects and RSA effects in the same region in the same cue condition. These findings suggest that, unlike RSC, cue unification could not be accomplished within a single entorhinal subregion. However, it remains an open question whether and how entorhinal subregions participate in the cue unification process.

When considering both fMRIa and RSA effects, our findings challenge the traditional dichotomy between landmark-based navigation and path integration within EC, that is, alEC and pmEC encode spatial information derived from landmarks and self-motion cues, respectively. Specifically, the right pmEC, which displayed fMRIa-based spatial coding in the self-motion high condition, also exhibited RSA-based spatial coding in the landmark low condition, although it remains uncertain whether this coding reflects unintended reliance on path integration. Similarly, in our earlier study, the left alEC demonstrated fMRIa-based spatial coding in the self-motion high condition ($t(21) = 2.071$, $p_{1\text{-tailed}} = 0.025$, $BF_{10} = 2.564$), although this effect did not survive the stringent multiple comparisons correction ($p_{corrected} = 0.177$ across eight tests; Chen et al., 2019, Supplemental Information). These findings align with the known intrinsic connectivity within the EC and its connections with the hippocampus (Van Strien et al., 2009).

Additionally, the hippocampus exhibited location-based RSA effects in the self-motion low condition, and the parahippocampal cortex (PHC) exhibited location-based RSA effects in the landmark high condition. However, these two effects were rather weak, with the Bayes factor indicating anecdotal evidence for the alternative null hypothesis or even the null hypothesis (BF_{10} = 1.581 in PHC and = 0.938 in the hippocampus). Both regions did not exhibit any fMRIa effects in the first place (ts < 0.4, $ps_{1\text{-tailed}} > 0.35$, $BFs_{10} < 0.32$).

Table S1. Location-based spatial information scores in Chen et al., 2019.
The scores were calculated for regions of interest in the medial temporal lobe. Significant scores are highlighted in BOLD. Because no ROIs exhibited significant RSA effects for both cue types simultaneously, the between-cue spatial information scores were not computed. alEC is short for anterior-lateral entorhinal cortex, and pmEC for posterior-medial entorhinal cortex.

Region	Condition	Mean	SE	T value	$P_{1\text{-tailed}}$	BF_{10}
Right alEC	Landmark high	-0.074	0.050	-1.481	0.923	0.100
	Landmark low	-0.067	0.034	-1.951	0.967	0.086
	Self-motion high	0.014	0.039	0.373	0.357	0.303
	Self-motion low	0.007	0.043	0.170	0.433	0.255
Right pmEC	Landmark high	-0.041	0.051	-0.796	0.782	0.135
	Landmark low	***0.089***	***0.037***	***2.413***	***0.013***	***4.588***
	Self-motion high	0.052	0.038	1.373	0.093	0.911
	Self-motion low	0.092	0.055	1.674	0.055	1.387
Left alEC	Landmark high	-0.011	0.057	-0.198	0.578	0.193
	Landmark low	-0.040	0.062	-0.637	0.266	0.147
	Self-motion high	0.061	0.039	1.553	0.068	1.166
	Self-motion low	0.003	0.047	0.063	0.476	0.234
Left pmEC	Landmark high	0.049	0.057	0.864	0.199	0.492
	Landmark low	-0.034	0.049	-0.705	0.755	0.142
	Self-motion high	0.020	0.040	0.511	0.308	0.343
	Self-motion low	0.056	0.040	1.394	0.089	0.938
Retrosplenial cortex	Landmark high	0.036	0.042	0.859	0.200	0.193
	Landmark low	0.031	0.048	0.662	0.258	0.147
	Self-motion high	0.067	0.051	1.312	0.102	1.166
	Self-motion low	0.051	0.042	1.216	0.119	0.234
Hippocampus	Landmark high	-0.023	0.039	-0.586	0.718	0.492
	Landmark low	-0.026	0.051	-0.519	0.695	0.142
	Self-motion high	-0.004	0.054	-0.082	0.532	0.343
	Self-motion low	***0.084***	***0.048***	***1.727***	***0.050***	***0.938***
Parahippocampal cortex	***Landmark high***	***0.087***	***0.049***	***1.762***	***0.047***	***1.581***
	Landmark low	0.014	0.044	0.315	0.378	0.288
	Self-motion high	0.052	0.055	0.958	0.175	0.547
	Self-motion low	0.002	0.040	0.052	0.480	0.232
Perirhinal cortex	Landmark high	-0.096	0.057	-1.677	0.945	0.910
	Landmark low	-0.072	0.039	-1.847	0.960	0.661
	Self-motion high	0.051	0.039	1.293	0.106	0.566
	Self-motion low	0.011	0.038	0.293	0.386	0.433

2.2. Re-examination of our second fMRI study (Chen et al., 2024)

We calculated both the location-based and response-based spatial information scores for the ROIs in the medial temporal lobe. The location-based scores were calculated using *GLM-RSA-location* (Table S2), while the response-based scores were calculated using *GLM-RSA-response* (Table S3). These two GLMs differed in how the events of interested were labeled and grouped. See the main text for detailed explanation of the construction of these two GLMs.

Table S2: Response-based spatial information scores in Chen et al., 2024.
The scores were calculated for regions of interest in the medial temporal lobe, using *GLM-RSA-response* (see the main text). Significant scores are highlighted in BOLD. Landmark# indicates results when statistical outliers have been winsorized. alEC is short for anterior-lateral entorhinal cortex, and pmEC for posterior-medial entorhinal cortex.

Region	Measurement	Mean	T value	$p_{1tailed}$	BF_{10}
Retrosplenial cortex	***Landmark***	***0.188***	***3.795***	***< 0.001***	***61.372***
	Landmark#	***0.172***	***4.233***	***< 0.001***	***146.819***
	Self-motion	***0.130***	***2.319***	***0.016***	***3.905***
	Between-cue	***0.146***	***3.143***	***0.003***	***17.248***
Hippocampus	***Landmark***	***0.118***	***2.254***	***0.018***	***3.504***
	Self-motion	***0.117***	***4.815***	***0.000***	***471.700***
	Between-cue	***0.125***	***3.147***	***0.003***	***17.362***
Parahippocampal cortex	***Landmark***	***0.153***	***3.420***	***0.001***	***29.334***
	Self-motion	***0.075***	***1.845***	***0.040***	***1.829***
	Between-cue	***0.100***	***1.979***	***0.031***	***2.249***
Perirhinal cortex	Landmark	0.085	1.251	0.113	0.804
	Self-motion	***0.144***	***3.212***	***0.002***	***19.652***
	Between-cue	***0.098***	***1.879***	***0.038***	***1.927***
Left alEC	Landmark	0.033	0.643	0.264	0.406
	Self-motion	0.081	1.456	0.081	1.050
	Between-cue	-0.020	-0.556	0.708	0.161
Right alEC	Landmark	0.030	0.533	0.300	0.365
	Self-motion	0.059	1.020	0.160	0.609
	Between-cue	***0.126***	***2.947***	***0.004***	***11.938***
Left pmEC	Landmark	0.041	0.839	0.206	0.497
	Self-motion	0.035	0.802	0.216	0.478
	Between-cue	***0.148***	***2.614***	***0.009***	***6.507***
Right pmEC	Landmark	0.037	0.751	0.231	0.453
	Self-motion	0.025	0.489	0.315	0.350
	Between-cue	0.050	1.019	0.160	0.608

Table S3. Location-based spatial information scores in Chen et al., 2024.
The scores were calculated for regions of interest in the medial temporal lobe, using *GLM-RSA-location* (see the main text). Significant scores are highlighted in BOLD.

Region	Measurement	Mean	T value	$p_{1tailed}$	BF_{10}
Retrosplenial cortex	***Landmark***	***0.193***	***3.207***	***0.002***	***19.486***
	Landmark#	***0.180***	***4.822***	***< 0.001***	***478***
	Self-motion	***0.094***	***1.938***	***0.034***	***2.109***
	Between-cue	0.063	1.014	0.162	0.604
Hippocampus	***Landmark***	***0.129***	***2.049***	***0.027***	***2.510***
	Self-motion	***0.096***	***2.444***	***0.012***	***4.833***
	Between-cue	0.035	0.867	0.198	0.513
Parahippocampal cortex	***Landmark***	***0.129***	***2.545***	***0.010***	***5.759***
	Self-motion	0.013	0.345	0.367	0.307
	Between-cue	0.044	0.808	0.215	0.481
Perirhinal cortex	Landmark	0.087	1.095	0.144	0.664
	Self-motion	0.069	1.435	0.084	1.021
	Between-cue	0.011	0.211	0.418	0.274
Left alEC	Landmark	0.040	0.635	0.266	0.403
	Self-motion	***0.101***	***2.007***	***0.030***	***2.348***
	Between-cue	-0.034	-0.739	0.766	0.146
Right alEC	***Landmark***	***0.111***	***1.938***	***0.034***	***2.108***
	Self-motion	-0.001	-0.015	0.506	0.230
	Between-cue	***0.071***	***1.773***	***0.046***	***1.642***
Left pmEC	Landmark	0.047	0.842	0.205	0.499
	Self-motion	0.032	0.712	0.243	0.435
	Between-cue	0.064	1.108	0.141	0.674
Right pmEC	Landmark	0.021	0.489	0.315	0.350
	Self-motion	0.009	0.190	0.426	0.270
	Between-cue	0.040	0.734	0.236	0.445

2.3. Searchlight analysis for our second fMRI study (Chen et al., 2024)

The searchlight analysis was conducted in each participant's native brain, using codes adapted from the TDT toolbox (Hebart et al., 2015) and a searchlight radius of 6 mm. At each step, for voxels within the searchlight, the response-based spatial information score was calculated in the same way as in the ROI-based analyses; the score was then assigned to the voxel at the center of the searchlight. The participant-specific brain maps of spatial information score were normalized to the Montreal Neurological Institute (MNI) template and then spatially smoothed with 3 mm isotropic FWHM. For the 2nd level analysis, we conducted directional one-sample t test against 0, using the nonparametric permutation approach (Nichols & Holmes, 2002). Cluster-level inference was used, with the customary cluster-defining threshold $T > 3$. We used the group-level anatomical mask comprising all our ROIs (MTL + RSC) for small volume correction.

As shown in Figure S1 and Table S4, RSC was involved in all three response-based RSA effects. For the response-based within-landmark spatial information score (Figure S1a), there was one significant cluster whose peaks were within the group-level anatomical mask of RSC ($k = 3202$, $p_{FWE,1\text{-tailed}} < 0.001$). Notably, one of the peak voxels (MNI [7, -46, 17]) was also a peak voxel within a significant cluster after multiple comparisons correction across the entire search volume ($k = 8999$, $p_{FWE,1\text{-tailed}} = 0.001$). In addition to this cluster, there were two significant clusters whose peaks resided in the fusiform gyrus and posterior parahippocampal gyrus.

For the response-based within-motion spatial information score (Figure S1b), there was one significant cluster whose peaks were either within the group-level anatomical mask of RSC or right at its edge ($k = 1416$, $p_{FWE,1\text{-tailed}} = 0.006$). Additionally, there were three significant clusters whose peaks resided in the hippocampus, fusiform gyrus, lingual gyrus, inferior temporal gyrus, and posterior parahippocampal gyrus. One of the peak voxels in the hippocampus (MNI [-33, -16, -18]) was also a peak voxel within a significant cluster after multiple comparisons correction across the entire search volume ($k = 1018$, $p_{FWE,1\text{-tailed}} = 0.029$).

For the response-based between-cue spatial information score (Figure S1c), there was one significant cluster whose peaks were right at the edge of the group-level anatomical mask of RSC ($k = 1901$, $p_{FWE,1\text{-tailed}} = 0.006$). Additionally, there was one significant cluster with peaks in the posterior hippocampus and posterior parahippocampal gyrus.

In brief, converging with the ROI-based analyses, the searchlight analysis demonstrated that RSC was involved in significant response-based spatial coding for both landmarks and self-

motion cues, which was also generalizable between the cue types in terms of response. These findings confirmed the robustness of the ROI-based analyses.

Beyond our pre-defined ROIs (Table S5 & Figure S2), we observed significant clusters with peaks in the middle temporal gyrus and precuneus for the response-based within-landmark spatial information score. For the response-based within-self-motion spatial information score, we observed significant clusters with peaks in the middle occipital gyrus, superior occipital gyrus, middle temporal gyrus, fusiform gyrus. For the between-cue spatial information score, we observed significant clusters with peaks in the middle occipital gyrus, middle temporal gyrus, and the fusiform gyrus.

For completeness, we also conducted the searchlight analysis for the three location-based spatial information scores. As shown in Figure S2, the pattern of results looked similar to that of the response-based spatial information scores. However, visual inspection reveals that the magnitudes of RSA effects were generally lower for the location-based than the response-based spatial information scores in most areas of the brain, including the posterior cingulate areas (including the RSC proper and the putative retrosplenial complex), precuneus, middle occipital gyrus, middle temporal gyrus, and angular gyrus. This disparity between response and location was more salient for within-motion and between-cue scores than the within-landmark score, because location and response was less dissociable in the landmark condition due to its high behavioral accuracy.

In summary, the searchlight results confirmed the validity of our earlier findings from the ROI-based analyses, demonstrating the presence of cue-independent spatial representations in RSC. Moreover, these spatial representations were generally tied more strongly to behavior than stimulus.

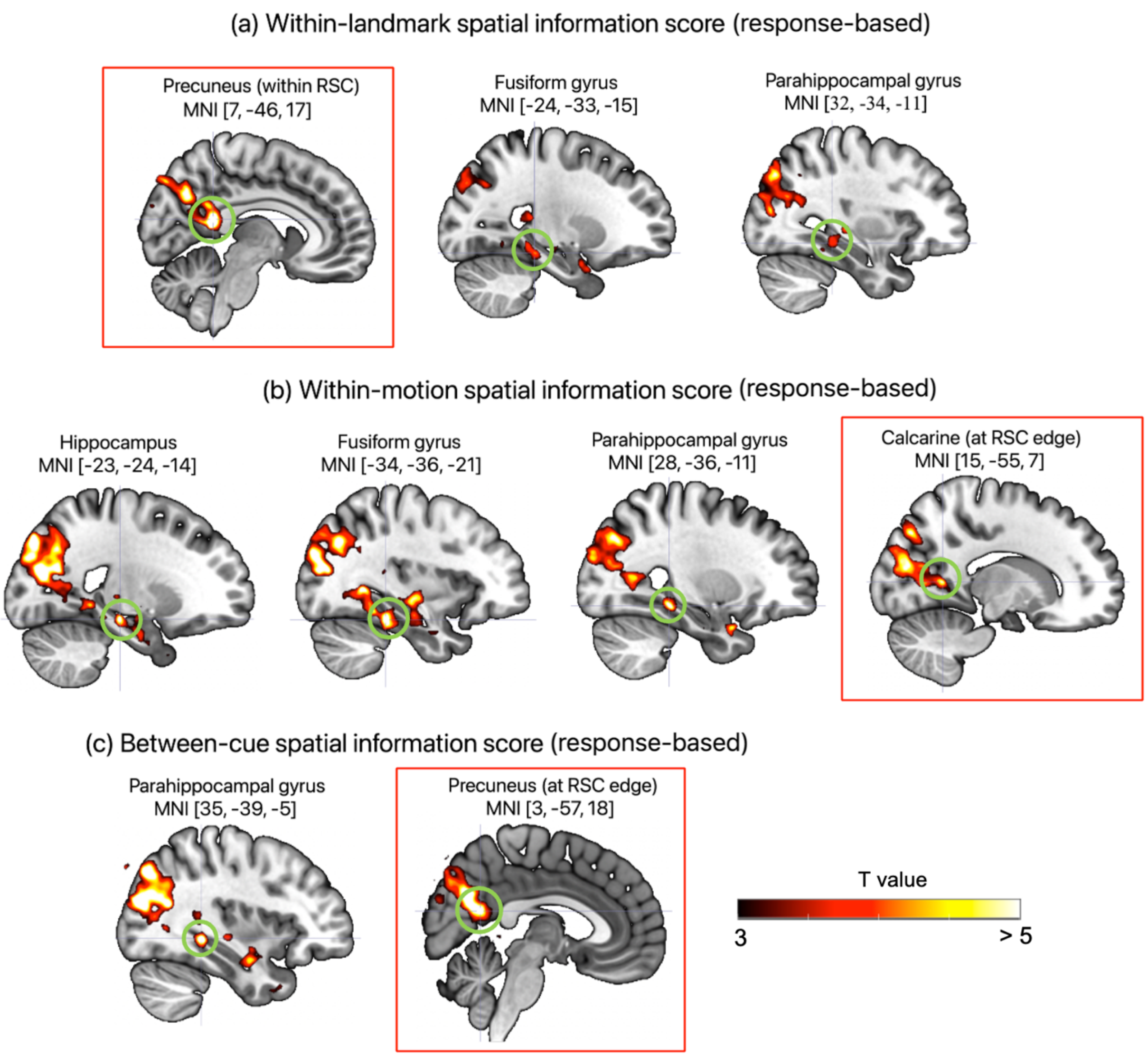

Figure S2. Searchlight analysis of response-based RSA effects.
For the 2nd level analysis, we conducted directional one-sample t test against 0, using the nonparametric permutation approach (Nichols & Holmes, 2002). Cluster-level inference was used, with the customary cluster-defining threshold T > 3. We used the group-level anatomical mask comprising all our ROIs (MTL + RSC) for small volume correction. Here, the parametric t maps are overlaid on the MNI template, thresholded at T > 3. Detailed results are listed in Table 1. The involvement of RSC is highlighted in red boxes.

Table S4. Searchlight analysis within pre-defined ROIs.
Multiple comparisons were corrected within the group-level anatomical mask consisting of all ROIs (bilateral MTL + bilateral RSC). We used nonparametric permutation-based tests with cluster-level inference (Nichols & Holmes, 2002). Cluster size (k) was determined at voxel-wise T > 3. In correspondence to Figure S1.

Brain Region	MNI		Voxel Level (T)	Cluster Size (k)	$p_{FWE\text{-}corr,\ 1\text{-}tailed}$
	RH	LH			
Within-landmark spatial information score (response-based)					
Precuneus (within RSC)	7, -46, 17		7.63	3202	< 0.001
Undefined (within RSC)		-2, -49, 20	5.43		
Precuneus (within RSC)		-7, -47, 11	5.31		
Fusiform gyrus		-24, -33, -15	4.20	1259	0.011
		-38, -28, -12	4.06		
Parahippocampal gyrus		-30, -40, -6	3.14		
Parahippocampal gyrus	32, -34, -11		3.97	831	0.022
Fusiform gyrus	26, -31, -18		3.18		
Parahippocampal gyrus	20, -38, -10		3.03		
Within-motion spatial information score (response-based)					
Undefined (close to hippocampus)		-23, -24, -14	6.08	2021	0.002
Fusiform gyrus		-38, -19, -24	4.51		
Hippocampus		-33, -16, -18	4.31		
Fusiform gyrus		-34, -36, -21	6.05	2425	0.002
Inferior temporal gyrus		-38, -34, -12	5.10		
Lingual gyrus		-21, -44, -5	4.85		
Parahippocampal gyrus	28, -36, -11		5.58	801	0.023
Fusiform gyrus	33, -42, -16		3.67		
Calcarine (at RSC edge)	15, -55, 7		5.08	1416	0.006
Precuneus (within RSC)		-7, -47, 15	4.97		
Calcarine (within RSC)		8, -53, 13	4.26		
Between-cue spatial information score (response-based)					
Parahippocampal gyrus	35, -39, -5		6.55	1284	0.015
Underfined (close to hippocampus)	22, -36, 10		4.68		
Parahippocampal gyrus	19, -40, -3		4.33		
Precuneus (at RSC edge)	3, -57, 18		5.96	1901	0.006
Precuneus (at RSC edge)		-11, -57, 19	5.79		
Calcarine (at RSC edge)	10, -57, 11		4.83		

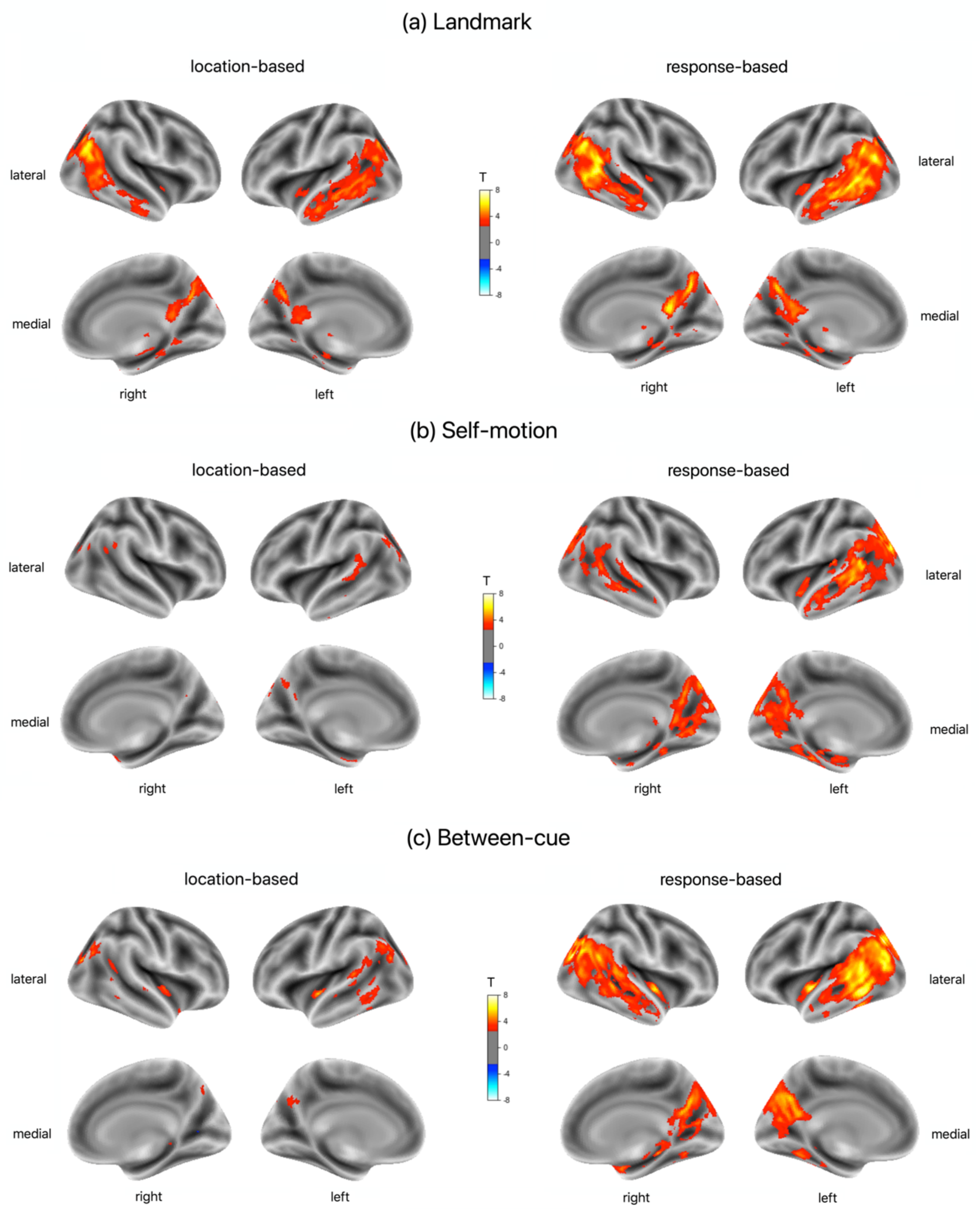

Figure S3. Location-based vs. response-based RSA effects in the entire search volume. The searchlight analysis was conducted for both the location-based and response-based RSA effects for each of the three cue conditions: within-landmark, within-motion, and between-cue. Here, the parametric t maps are projected to the standard brain's surface, thresholded at T > 3.

Table S5. Searchlight analysis in the entire search volume.
Multiple comparisons were corrected across the entire search volume. We used nonparametric permutation-based tests with cluster-level inference (Nichols & Holmes, 2002). Cluster size (k) was determined at voxel-wise T > 3. In correspondence to Figure S2.

Brain Region	MNI		Voxel Level (T)	Cluster Size (k)	$p_{FWE\text{-}corr,\ 1\text{-}tailed}$
	RH	LH			
Within-landmark spatial information score (response-based)					
Middle temporal gyrus	58, -63, 7		9.34	24288	0.0002
	45, -52,16		8.51		
	45, -67, 29		8.01		
Middle temporal gyrus		-62, -47, -2	8.94	37369	0.0002
		-50, -64, 21	8.47		
		-57, -53, 3	7.56		
Precuneus	3, -67, 34		7.63	8999	0.0010
	7, -46, 17		7.63		
	2, -59, 29		5.95		
Within-motion spatial information score (response-based)					
Middle occipital gyrus		-28, -79, 17	8.31	43526	0.004
Superior occipital gyrus		-25, -85, 39	8.05		
Middle temporal gyrus		-49, -50, 10	6.68		
Middle temporal gyrus	33, -62, 7		6.80	2435	0.0082
	41, -56, 4		4.91		
	56, -57, 8		4.85		
Fusiform gyrus		-34, -36, -20	6.06	2368	0.0084
		-34, -53, 1	5.88		
		-39, -34, -11	5.38		
Hippocampus		-36, -14, -6	5.84	1018	0.0294
		-33, -16, -18	4.31		
		-27, -18, -6	4.17		
Superior temporal gyrus	55, -14, -6		5.82	1698	0.0160
Middle temporal gyrus	55, -27, 0		5.63		
	64, -34, -11		5.37		
Middle temporal gyrus		-49, 2, -22	5.06	1205	0.0246
		-44, -6, -16	5.06		
		-53, -13, -19	4.47		
Between-cue spatial information score (response-based)					
Middle occipital gyrus	32, -83, 20		10.55	102705	0.0002
		-31, -69, 31	9.69		
Undefined (close to middle temporal gyrus)		-44, -15, -11	9.04		
Fusiform gyrus		-35, -52, -6	5.54	823	0.0376
		-27, -53, -9	4.43		

3. Simulating fMRIa- and RSA-based spatial coding

Methods. We simulated activations of individual neurons in response to the location occupation event (Figure 1b, Phase 4). Each neuron had a preferred location, but could also respond to other locations: the narrower the neuronal tuning curve and the more distant of the occupied location to the neuron's preferred location, the weaker the neuron's activation level. There were four types of neurons, each type preferably responding to one of the four test locations. We manipulated two parameters: the width of neuronal tuning curve (σ_{tc}) and the evenness of spatial distribution of neurons across voxels (σ_{vd}). Larger values of σ_{tc} correspond to more widespread neuronal tuning curves, and thus, larger **representational overlap among different locations at the neuronal level**. Larger values of σ_{vd} correspond to more uneven distribution of neurons across voxels, and thus **coarser spatial clustering of neurons**. Note that σ_{vd} was manipulated for each neuron type. When the across-voxel distribution of neurons was uneven, the number of such neuron contained in a voxel differed vastly among voxels. When the across-voxel distribution of neurons was even, the number of such neuron contained in a voxel was similar among voxels.

We simulated 100 voxels. Each voxel contained 1000 neurons, with equal numbers of neurons preferring each of the four test locations (i.e., 250 neurons for each location). Each neuron had its own tuning curve, which was a Gaussian distribution ($N(\mu_{tc}, \sigma_{tc})$). The mean of the tuning curve corresponds to its preferred test location (μ_{tc}), meaning the neuron is activated most strongly when the occupied location coincides with its preferred test location. The width of the neuronal tuning curve is reflected in its standard deviation (σ_{tc}): the more widespread the curve, the less specific the neuron responds to various spatial locations. The activation of a given neuron (a) is determined by the relative position of the occupied location (x) to the tuning curve:

$$a(x) = \frac{1}{\sqrt{2\pi}\sigma_{tc}} e^{-\frac{(x-\mu_{tc})^2}{2\sigma_{tc}^2}}$$

Note that the parameter σ_{tc}, which reflects representational overlap among test locations at the neuronal level, was manipulated.

Another parameter to manipulate is σ_{vd}, the standard deviation of the voxel-to-voxel distribution of the location-sensitive neurons. However, we did not directly manipulate this parameter. We assumed that the proportion of neurons falling in a specific voxel obeyed a normal

distribution ($N(0, \sigma_0)$). We then normalized the proportions of neurons across voxels so that their sum equaled to 1. Hence, the larger the σ_0, the more even the voxel-to-voxel distribution of neurons, and thus the smaller the σ_{vd}. σ_{vd} was then calculated in a post hoc manner after determining the number of neurons in each voxel.

To model the location-based fMRIa effects, we assumed the following neuronal adaptation rule. Let x_{i-1} and x_i represent the location visited by the navigator in the preceding trial and the current trial, respectively. Let $a_{0,i-1}$ and $a_{0,i}$ represent the activation level of a given neuron for these two locations, respectively, when no adaptation occurs. Let a_i represent the activation level for the current trial with adaptation, with an adaptation rate (r) of 0.5. These values are expressed as:

$$a_{0,i-1}(x) = \frac{1}{\sqrt{2\pi}\sigma_{tc}} e^{-\frac{(x_{i-1}-\mu_{tc})^2}{2\sigma_{tc}^2}}$$

$$a_{0,i}(x) = \frac{1}{\sqrt{2\pi}\sigma_{tc}} e^{-\frac{(x_i-\mu_{tc})^2}{2\sigma_{tc}^2}}$$

$$a_i = \max\left(a_{0,i}(x) - \left(a_{0,i-1}(x) \times r\right), 0\right)$$

The activation level of a voxel equaled to the sum of activation levels of all neurons within the voxel. Subsequently, location-based RSA and fMRIa effects were calculated using the same methods as in the main fMRI analyses in the current and our previous studies (Chen et al., 2019, 2024), based on voxel activation levels. For each effect type, we calculated two scores: distance score and location identity score. To calculate the location identity score, we only considered whether the two locations were the same (= 0) or different (= 1). To calculate the distance score, we used the continuous inter-location distance variable, with pairs of same locations excluded (i.e., distance = 4 m, 8 m, or 12 m).

Results. Figure S4 depicts the distance and location identity scores as a function of the two manipulated parameters (σ_{tc}, σ_{vd}), separately for fMRIa and RSA effects. There are three key findings. First, these two parameters influenced fMRIa- and RSA-based coding in different

manners. Second, these two parameters also influenced location identity coding and distance coding in different manners. Finally, both fMRIa- and RSA-based distance coding required a certain degree of neuronal representational overlap, as reflected in the parameter σ_{tc}.

Evenness of across-voxel distribution of neurons (σ_{vd}). For the fMRIa-based coding, influence of σ_{vd} was relatively limited. When the across-voxel neuron distribution was extremely uneven (i.e, σ_{vd} is very large (> 340), a & b), both the location identity coding and distance coding were relatively diminished. This parameter did not impact the two types of fMRIa-based coding unless it is extremely large (< 250).

On the contrary, for the RSA-based location identity coding and distance coding, the influence of σ_{vd} was more continuous (c&d): the more even the across-voxel neuron distribution, the weaker the coding. Intriguingly, the influence of this parameter on RSA-based coding was in a direction opposite to its influence on fMRIa-based coding (a & b).

Width of neuronal tuning curve (σ_{tc}). The width of neuronal tuning curve influenced both fMRIa- and RSA-based coding in similar ways (a&c, b&d). For the location identity coding, the broader the neuronal tuning curve, the weaker the coding (b&d). On the contrary, for the distance coding, when the neuronal tuning curve was very sharp, the coding approached zero, which resonates with our earlier observation of fMRIa-based coding of location identity in RSC for landmarks (Chen et al., 2024). When the tuning curve was not too sharp, σ_{tc} affected distance coding and location identity coding in similar ways: the broader the neuronal tuning curve, the weaker the coding (a&c). In other words, for both RSA and fMRIa effects, the emergence of distance coding requires a certain degree of overlap in neuronal representation. In our studies, we observed distance coding for both cue types in terms of RSA and for self-motion cues in terms of fMRIa.

In summary, the simulation findings demonstrate that the width of neuronal tuning actually influences fMRIa-based coding and RSA-based coding in similar ways, contradicting the tuning-versus-clustering hypothesis proposed by Drucker & Aguirre (2009, Cerebral Cortex). On the contrary, the evenness of the across-voxel distribution of neurons influences these two types of coding in quite different ways. Showing that MVPA effects benefit from relatively coarse spatial distributions of neurons across a brain region, our simulation results are consistent with the tuning-versus-clustering hypothesis. Taken together, our simulation results are partially consistent with the tuning-versus-clustering hypothesis. Discrepancies between our simulation results and Drucker

& Aguirre's hypothesis are caused by the fact that across-voxel distribution of neurons is not considered in Drucker & Aguirre's hypothesis, while fMRI measures voxel-wise signals.

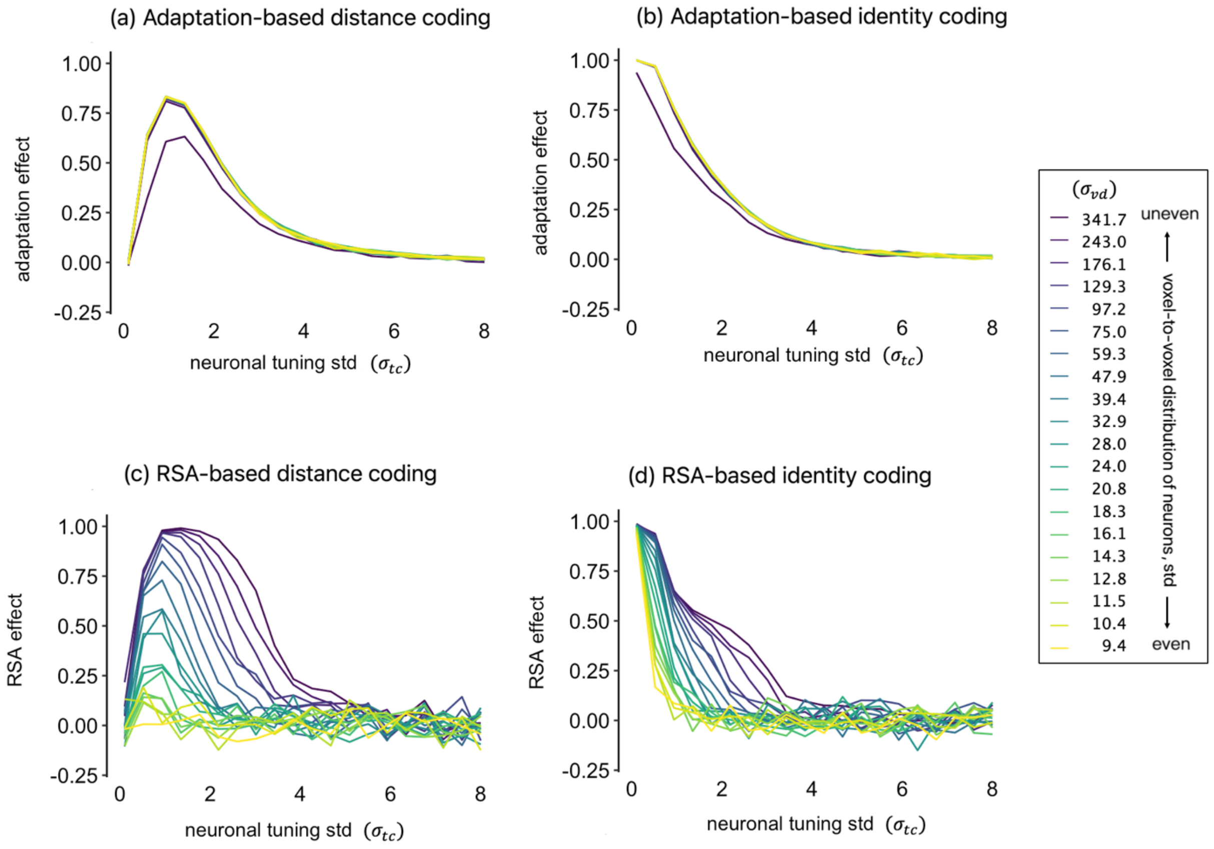

Figure S4. Simulating location-based fMRIa and RSA effects

We investigated influences of neuronal tuning curve and voxel-to-voxel distribution of neurons on fMRIa-based (a&b) and RSA-based spatial coding (c&d). Two different types of coding are displayed: distance coding (a&c) and location identity coding (b&d). in each plot, the horizontal axis represents the standard deviation of the neuronal tuning curve (σ_{tc}), the vertical axis represents the magnitude of the coding, and lines of different colors correspond to different evenness of spatial distribution of neurons across voxels (σ_{vd}).
